# BMP antagonist CHRDL2 enhances the cancer stem‐cell phenotype and increases chemotherapy resistance in colorectal cancer

**DOI:** 10.1002/1878-0261.70064

**Published:** 2025-05-28

**Authors:** Eloise Clarkson, Annabelle Lewis

**Affiliations:** ^1^ Division of Biosciences, Department of Life Sciences, College of Health and Life Sciences, Centre for Genome Engineering and Maintenance Brunel University London Uxbridge UK

**Keywords:** bone‐morphogenic protein, cancer stem cell, chordin‐like 2, colorectal cancer, WNT signalling pathway

## Abstract

Bone morphogenetic protein (BMP) antagonists have been increasingly linked to the development of colorectal cancer (CRC). BMP signalling operates in opposition to the WNT signalling pathway, which sustains stem‐cell maintenance and self‐renewal of the normal intestinal epithelium. Reduced BMP and elevated WNT signalling lead to expansion of the stem‐cell compartment and the hyperproliferation of epithelial cells, a defining characteristic of CRC. Chordin‐like‐2 (CHRDL2) is a secreted BMP antagonist, with overexpression linked to poor prognosis and variants in the gene shown to be associated with an elevated CRC risk. However, the detailed mechanism by which CHRDL2 contributes to CRC is unknown. In this study, we explored the impact of CHRDL2 overexpression on CRC cells to investigate whether CHRDL2's inhibition of BMP signalling intensifies WNT signalling and enhances the cancer stem‐cell phenotype and response to treatment. Our research approach combines 2D cancer cell lines engineered to inducibly overexpress CHRDL2 and 3D organoid models treated with extrinsic CHRDL2, complemented by RNA sequencing analysis. CHRDL2 was found to enhance the survival of organoids and CRC cells during chemotherapy and irradiation treatment due to activation of DNA damage response pathways. Organoids treated with secreted CHRDL2 exhibited elevated levels of stem‐cell markers and reduced differentiation, as evidenced by diminished villi budding. RNA‐seq analysis revealed that CHRDL2 increased the expression of stem‐cell markers, WNT signalling and other well‐established cancer‐associated pathways through BMP inhibition. These findings collectively suggest that CHRDL2 overexpression could affect response to CRC therapy by enhancing DNA repair and the stem‐cell potential of cancer cells, and its role as a biomarker should be further explored.

## Graphical abstract

Overexpression of *CHRDL2* in colon cancer cells makes them more stem‐like and resistant to chemo‐ and radiotherapy. CHRDL2‐high cells have upregulation of the WNT pathway, genes involved in the DNA damage response (DDR) pathway and epithelial‐to‐mesenchymal transition (EMT). This leads to quicker repair of damaged DNA and more cell migration.

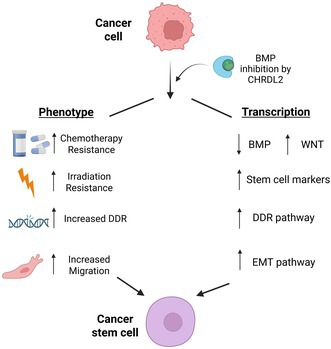

**Abbreviations**
ATM: Ataxia‐telangiectasia mutated
BMI1: B lymphoma Mo‐MLV insertion region 1 homologue
BMP: bone‐morphogenic protein
CHRDL2: Chordin‐like 2
CRC: colorectal cancer
CSC: cancer stem cell
DSB: double‐stranded break
ISC: intestinal stem cell
LGR5: leucine‐rich repeat‐containing G‐protein coupled receptor 5
LGR6: leucine‐rich repeat‐containing G‐protein coupled receptor 6
WNT: WNT signalling pathway
γH2AX: H2A histone family member X

## 1 Introduction

Colorectal cancer (CRC) ranks as the third most prevalent cancer globally, with over 1 million reported cases in 2020 by GLOBOCAN [1]. Originating from mutations within intestinal epithelial cells, CRC leads to the formation of polyps, adenocarcinomas and eventually metastatic cancer. Although many signalling pathways are disrupted in CRC, the WNT/β‐catenin signalling pathway is the most commonly affected, overactive in nearly all CRC cases [2].

WNT signalling is the fundamental pathway regulating intestinal stem‐cell (ISC) proliferation and fate. WNT activation is localised at the base of the intestinal crypt, where it controls ISC fate and renewal, a vital component of intestinal maintenance [3]. Cryptal ISCs are organised hierarchically, with rapidly proliferating ISCs residing at the bottom of crypts and more slowly proliferating or regenerative ISCs slightly displaced from the base of the crypt at the +4 position [4, 5]. A third more mature subset of rapidly proliferating cells migrates further up the crypt into the transit amplifying (TA) zone. ISCs are identified by the expression of the LGR5+ marker, and slow‐cycling cells in the +4 position have previously been identified by the presence of the BMI1+ marker, as well as HPOX and TERT, but with conflicting data about the specific markers and role for these cells [6].

WNT signalling transduces by sequestering the β‐catenin destruction complex, raising intracellular β‐catenin levels, and activating stem‐cell and oncogenic pathways in stem‐cell crypts [7, 8]. Disruption of WNT signalling eliminates progenitor phenotypes within the crypts and results in crypt loss [9]. Despite the need for WNT signalling to maintain the intestinal lining and crypt formation, sustained or elevated WNT signalling can cause hyperproliferation and oncogenic transformation in ISCs [10]. WNT signalling has been shown to work in a counter gradient to BMP signalling, which is found in the intestinal villi and promotes cellular differentiation and maturation [10, 11, 12]. These gradients of BMP and WNT signalling are a major controlling factor in crypt‐villi architecture and intestinal homeostasis.

In contrast to WNT signalling, BMP signalling is localised in the differentiated compartment of the crypt/villi and aids in cellular differentiation, proliferation and migration [13]. BMPs have been shown to have paradoxical effects in cancer, with specific ligands acting to inhibit and promote tumorigenesis in different tissues and contexts [14, 15, 16, 17]. BMPs belong to the TGF‐β superfamily and bind to a complex of transmembrane serine‐threonine kinase receptors I and II (BMPRs I and II) [18]. This initiates phosphorylation of the type I receptor by the type II receptors, triggering phosphorylation of a receptor‐associated SMAD that subsequently complexes with SMAD4 and translocates to the nucleus to regulate gene transcription [19]. While epithelial and mesenchymal cells express BMPs and their receptors, BMP antagonists are primarily found in the mesenchyme. In the intestine, they are largely expressed by intestinal cryptal myofibroblasts and smooth muscle cells. These antagonists block BMP signalling in the stem‐cell compartment, maintaining high levels of WNT signalling and therefore the stem cells [11].

BMP antagonists can bind directly to BMPs or their receptors [20]. Some well‐studied BMP antagonists include Noggin, which has been implicated in promoting skin and breast cancer tumorigenesis, and the Gremlins (GREM1 and 2), with repression of GREM1 shown to inhibit tumour cell proliferation. The Chordin family of proteins have also been implicated in CRC, including Chordin, Chordin‐like 1 (CHRDL1) and Chordin‐like 2 (CHRDL2) [21, 22, 23, 24]. One of the best‐studied BMP antagonists, Noggin, has previously been shown to inhibit BMP signalling in a mouse model, resulting in the formation of numerous ectopic crypts perpendicular to the crypt‐villus axis [12]. Similarly, overexpression of GREM1 in Hereditary mixed polyposis syndrome (HMPS) leads to the persistence or reacquisition of stem‐cell properties in LGR5‐negative cells outside the stem‐cell niche. Ectopic crypts, enhanced proliferation and intestinal neoplasia [25]. Together, this suggests that abolition of BMP signalling through its antagonists leads to the formation of stem‐like qualities in intestinal epithelial cells, leading to oncogenic transformation.

CHRDL2 is a BMP antagonist which prevents BMP ligands 2, 4 and 6, from interacting with their cognate cell‐surface receptors [22, 26]. CHRDL2 has been shown to bind directly to BMPs, preventing signalling though phospho SMAD1/5. Furthermore, CHRDL2 inhibits the effects of BMP signalling on proliferation inhibition and apoptosis [27]. CHRDL2 mRNA upregulation has been observed in colon, breast, liver and prostate cancer [27, 28] and high levels predict poor prognosis and correlate with increased tumour size and later TNM stages [27]. CHRDL2 has been highlighted as a potential circulating protein biomarker for CRC, in which genetically predicted higher levels of CHRDL2 were associated with an increased risk of CRC [29]. CHRDL2's precise functional role in these cancers is not always clear but it has been shown to increase cellular proliferation, migration and invasion in osteosarcoma cell lines by regulation of the PI3k/AKT pathways through binding to BMP9 [30]. However, the role of BMP signalling in cancer, and therefore, the effect of BMP inhibition by CHRDL2 in cancer remains poorly characterised.

In this study, we used CRC cell lines engineered to stably overexpress CHRDL2 in an inducible manner, to investigate the cellular and transcriptional pathways activated by CHRDL2 expression and BMP inhibition. We have shown that CHRDL2 has measurable effects on cell proliferation and significantly changes the response to DNA‐damaging chemotherapy. To gain deeper insights into CHRDL2's role in stem‐cell maintenance and differentiation, we cultivated 3D intestinal organoids supplemented with secreted forms of CHRDL2. Collectively, our findings suggest that CHRDL2 modulates stem‐cell pathways in CRC, potentially impacting the response to common chemotherapeutic interventions.

## 2 Materials and methods

### 2.1 Cell culture and maintenance

Immortalised human colorectal adenocarcinoma cell lines Caco‐2 (RRID:CVCL_0025), COLO 320 (RRID:CVCL_1989), LS180 (RRID:CVCL_0397) and RKO (RRID:CVCL_0504) were acquired from ATCC and authenticated by STR analysis (Eurofins, Ebersberg, Germany) within 3 years of use for this study. Cell‐line morphology was continually checked to ensure authenticity. All experiments were performed with mycoplasma‐free cells. Cells were maintained in Gibco Dulbecco's modified Eagle medium (DMEM) (Merck, Gillingham, UK) supplemented with 10% fetal bovine serum (FBS) (Merck), and 1% penicillin–streptomycin (Merck). Cells were grown in a humidified atmosphere at 37 °C with 5% CO_2_. Subculturing was performed every 72 h to maintain a cell confluency of <80%.

### 2.2 Generation and validation of CHRDL2 overexpressing cell lines

CHRDL2 full‐length cDNA (Genecopoeia GC‐H1938, Caltag Buckingham, UK) was cloned into pCW57.1 (Addgene #41393, Watertown, MA, USA) using Gateway technology (Invitrogen, Thermo Fisher, Waltham, MA, USA), followed by validation by Sanger sequencing and restriction digest. The vector was then transfected into HEK293 cells along with viral packaging vectors (2nd generation system—pCMV‐dR8.2 and pCMV‐VSV‐G) using Lipofectamine 2000 (Invitrogen, Fisher Scientific, Loughborough, UK). Virus‐containing media was collected, sterilised and titres measured (Go‐Stix, Takara Bio, London, UK). The cell lines CACO2, COLO320, LS180 and RKO were transduced, and cells with integrated pCW57.1‐CHRDL2 were selected with puromycin. To confirm overexpression, doxycycline was added at (0.1 μg·mL^−1^, 1 μg·mL^−1^ (CHRDL2+) or 10 μg·mL^−1^ (CHRDL2++) RNA was extracted (RNeasy, QIAGEN, Manchester, UK) and quantified by real‐time reverse transcriptase polymerase chain reaction (qPCR) using TaqMan technology (Hs00248808_m1) according to the manufacturer protocol (Applied Biosystems, Fisher Scientific, Loughborough, UK). Each assay was repeated in triplicate.

### 2.3 Western blot

For intracellular protein detection, cells were lysed by resuspension in RIPA buffer. For secreted protein expression, cells given doxycycline expression at 10 mg·mL^−1^ were incubated for 72 h. Media was collected and concentrated through Amicon^®^ Ultra centrifugal filters (Merck) with a pore size of 30 kDa. Media was then diluted 1:25, 1:25 and 1:100 with RIPA buffer. For intracellular proteins, 30 μg of protein were loaded per sample. Protein samples were separated via 4–12% sodium dodecyl sulfate polyacrylamide gel electrophoresis under denaturing conditions and then transferred onto the nitrocellulose membrane (Millipore, Merck, Gillingham, UK) under 20 V. Membranes were blocked with 5% milk for 1 h at room temperature. Membranes were then incubated with primary antibody in TBST‐5% BSA overnight at 4 °C. Membranes were then washed with TBST. Secondary antibody was added for 1 h at room temperature. Membranes were imaged through incubation with Enhanced chemiluminescence (ECL). The ratio of optical density of the bands was measured by a gel image analysis system (Bio‐Rad, Watford, UK) and normalised to B‐actin as a loading control. For extracellular protein detection, ponceau stains were quantified and used as a loading control. The following antibodies were used: CHRDL2 (1:1000, Catalogue number: AF2448, Bio‐techne, Abingdon, UK), P‐SMAD1/5 (1:1000. Catalogue number: #9516, Cell Signalling, Danvers, MA, USA), Goat anti‐mouse (1:2000. Catalogue number: ab205719, Abcam, Cambridge, UK), Goat anti‐rabbit (1:5000. Catalogue number: p0448, Dako, Agilent, Stockport, UK).

### 2.4 Cell proliferation assay

To assess cellular proliferation during CHRDL2 overexpression, cells were plated at a density of 5 × 10^3^ cells per well in 96‐well plate with 8 replicates per condition: DMSO control, CHRDL2+ or CHRDL2++ treatments. Cellular proliferation was assessed via MTS assay (CellTiter 96^®^ Promega, Southampton, UK) at 24, 48 and 72 h.

### 2.5 Flow cytometry

COLO320 cells were plated in six‐well plates at a density of 1 × 10^5^ cells/well under standard media conditions, supplemented with DMSO or CHRDL2++ treatments. For chemotherapy flow cytometry analysis, cells were treated with 25 μm Oxaliplatin. Cells were grown for 48 h before harvesting by trypsinisation and washed once with cold PBS. To investigate cell‐cycle progression, cells were resuspended in either a PBS control or Hoechst 33 342 (62 249; Thermo Scientific, Fisher Scientific, Loughborough, UK) diluted in PBS and incubated at 37 °C for 2 h with slight agitation. Finally, samples were pelleted and resuspended in PBS, and flow cytometry analysis was performed using the ACEA Novocyte system (Agilent). The percentages of cells in phases of the cell cycle were analysed through Novocyte software. To investigate the proportion of Ki67+ cells, cells were incubated with Ki67 BV711 (407‐5698‐80; Thermo Scientific) for 20 min prior to analysis. To investigate apoptosis, cells were stained with Zombie Aqua (423 101; BioLegend, London UK) and annexin‐V antibody (V13242, Thermo Scientific) for 30 min each at room temperature. Subsequently, cells were washed once with PBS and analysed by flow cytometry using the manufacturer's software.

### 2.6 Colony formation assay

To assess the ability of single cells to generate colonies and cell survival ability, a clonogenic assay was performed. Cells were plated at 100 cells per well of a 6‐well plate. Cells were treated with doxycycline treatment as before in varying concentrations of 10 μg·mL^−1^, 1 μg·mL^−1^ and 0.1 μg·mL^−1^ or DMSO. Plates were incubated for 2 weeks until visible colonies were formed. Every 72 h, doxycycline and DMSO treatments were refreshed. After 2 weeks, cells were fixed with >98% methanol at −20 °C and stained with crystal violet stain (0.5%, in 20% methanol). Colonies were counted through ImageJ.

### 2.7 Drug dose–response assay

To assess the ability of cell lines to withstand treatment from commercial chemotherapy drugs, a drug dose–response curve was performed. Chemotherapy drugs used were as follows: 5‐fluorouracil (5FU), irinotecan and oxaliplatin (Merck). A serial dilution was performed in standard cell culture media to give a range of concentrations (5FU 0–10 000 μm, oxaliplatin 0–4000 μm, irinotecan 0–500 μm). Cells from culture were seeded on a 96‐well plate at a density of 2 × 10^5^ in 100 μL standard media with 10 mg·mL^−1^ doxycycline and incubated for 24 h. PBS was added in the surrounding wells to prevent evaporation of media. The media was then aspirated, and 100 μL of the diluted drug with 10 mg·mL^−1^ doxycycline was added to the corresponding well and incubated for 72 h. An MTS assay (CellTiter 96^®^ Promega) was then performed as above to measure the numbers of surviving cells present. Results were analysed using GraphPad Prism. Nonlinear regression was used to calculate IC50 values.

### 2.8 Radiation

To assess the effect of radiation on our CHRDL2 overexpressing cells, RKO cells were plated at a density of 1 × 10^5^ in 10 cm round dishes and treated with DMSO control or CHRDL2++ doxycycline treatment. After 24 h, cells were irradiated using x‐ray irradiation at 0 GY, 2 GY, 4 GY and 6 GY. The media and CHRDL2++ treatment were refreshed, and cells were incubated under standard conditions for a further 48 h. Subsequently, cell number was counted using Trypan blue cell viability assay (T10282, Thermo Scientific, Fisher Scientific).

### 2.9 Immunofluorescence

For cellular protein detection, cells were plated on coverslips and grown to ~70% confluency. Cells were fixed with methanol, and the cellular membrane was permeabilised with TRITON × 0.5% for 5 min. Cells were blocked with 1% BSA for 1 h at 37 °C, and then incubated with primary antibodies in 1% BSA for 1 h at 37 °C. A secondary antibody was then added to cells for 1 h at 37 °C. 5 μL of mounting media with DAPI (VECTASHIELD® WZ‐93952‐27; Cole‐Parmer, St. Neots, UK) was then placed onto the coverslips, and coverslips were fixed onto slides for imaging. Images were obtained using a Leica DM4000 system, and corrected total cell fluorescence was obtained using ImageJ. The following antibodies were used: H2AX (1:50; Catalogue number: ab195188, Abcam), ATM (1:50; Catalogue number: ab2354, Abcam), P53 (1:800. Catalogue number: 25275, Cell Signalling), B‐catenin (1:100. Catalogue number: 610154, BD biosciences, CA, USA), Ki67 (1:100. Catalogue number: D3B5, Cell signalling), Rad21 (1:100. Catalogue number: ab992, Abcam), Ku70 (1:100. Catalogue number: 2172, Abcam), Alexa Fluor Goat anti‐mouse AF488 (1:50. Catalogue number: A‐11001, Abcam) and Alexa Fluor Goat anti‐rabbit AF568 (1:50. Catalogue number: A‐11011, Abcam).

### 2.10 Comet assay

COLO320 cells were plated at a density of 1 × 10^5^ in with DMSO control or CHRDL2++ doxycycline and treated with IC50 oxaliplatin. After 72 h, cells were harvested and diluted in PBS at a density of 10^4^/mL. Cell suspension was mixed 1:5 with low‐gelling agarose, and 100 μL was placed on Polylysine coated slides dipped in 1% agarose (Merck) and allowed to set. A further 100 μL low‐gelling agarose (Merck) was placed on top and allowed to set. Cell lysis was then performed by submerging slides in lysis buffer (2% N‐lauroylsarcosine sodium salt (Merck), 0.5 M NA_2_EDTA (Merck) and 0.1 mg·mL^−1^ proteinase K) for 1 h. Slides were then washed in Electrophoresis buffer for 1.5 h (90 mm Tris Buffer (Fisher Fisher Scientific, Loughborough, UK), 90 mm boric acid (Merck) and 2 mm NA_2_EDTA). Slides were placed in an electrophoresis tank and submerged in electrophoresis buffer, under 20 V current for 40 min. Slides were then stained with 1% SYBR SAFE (Invitrogen, Fisher Scientific) in TBE for 20 min, before dehydration through submersion in 70%, 90% and 100% ethanol. Slides were visualised by Leica DM4000 system, and tails were measured using ImageJ.

### 2.11 Organoid preparation, culture and maintenance

Organoids were maintained in a humified atmosphere at 37 °C with 4% CO2. Organoids were grown in ADF media as described: Advanced DMEM/F12, 2 mm GLUTAMAX, 1 mm N‐acetylcysteine and 10 mm HEPES, supplemented with 1% PS, 10% B27 and 5% N2. Growth factors were also given to the media surrounding the basement membrane extract (Cultrex, Bio‐Techne, Minneapolis, MN, USA): 1% Mouse recombinant Noggin (PeproTech, London, UK), 1% mouse recombinant EGF (Invitrogen, Fisher Scientific) and 5% Recombinant human R‐spondin (PeproTech, London, UK)).

Organoids were generated from wild‐type mice (C57BL6/J, acquired from Charles River, Cambridge, UK) of both sexes aged between 6 and 12 weeks. Mice were housed together in individually ventilated cages supplied with bedding, enrichments and *ad libitum* access to food and water. All procedures were carried out in accordance with the UK Home Office ‘Animals (Scientific Procedures) Act 1986’ and with approval from the Brunel University Animals Welfare and Ethical Review Board and under the personal, project and establishment licences I0578EED1, PDF0B94C3 and XEC0493FD, respectively. Briefly, crypts were isolated from murine small intestine and washed with PBS. Villi and differentiated cells were scraped off intestine using a glass microscope slide. Sections of intestine were cut into 2 mm segments and transferred to ice‐cold PBS. Pipettes were coated in FBS, and intestinal segments were washed through pipetting up and down to dislodge single cells and debris. PBS was removed, and washes were repeated 5 times. Segments were then resuspended in 2.5 mm EDTA/PBS to loosen crypts and rotated at 4 °C for 30 min. The supernatant was then removed, and segments were resuspended in ADF media. The entire volume was pipetted up and down several times, and then, the supernatant removed and centrifuged for 5 min at 1200 rpm at 4 °C. The supernatant was removed, and the resulting pellet was resuspended in 10 mL ADF media and passed through a 70 μm cell strainer into a clean 15 mL falcon tube. The tube was then centrifuged for 2 min at 600 rcf at 4 °C so that single cells will not be included in the pellet, and the supernatant was removed. This was repeated 3 times. Finally, the pellet was resuspended in 50 μL ADF media and 100 μL Cultrex, and pipetted 40 μL/well. Passaging of organoids was repeated every 48 h and consisted of transferring organoid to a 15 mL conical tube, pipetting up and down to break up organoids. Organoids were then centrifuged for 2 min at 600–800 rpm at 4 °C and then resuspended in ADF with Cultrex as described previously.

### 2.12 Organoid chemotherapy treatment

Organoids were plated in triplicate in a 24‐well plate and treated with 5FU at 0.5, 1 and 5 μm 5FU. After 96 h, images were taken of each well, and the number of live organoids was counted blind by an independent researcher.

### 2.13 Organoid immunofluorescence staining

Organoid samples were prepared for staining by removal of growth media and pelleted through centrifugation at 600 ***g***. Organoids were then fixed through resuspension in 500 μL neutral buffered formalin (Merck) for 10 min, before pelleting at 400 g and resuspension in 70% ethanol for 1 min. Organoids were then pelleted at 400 g and resuspended in 50 μL of low‐gelling agarose (Merck) and incubated on ice for 30 min, before embedding in paraffin blocks using standard protocols. Sectioning of organoids was performed at 5 μm through standard microtome sectioning and left to dry on slides.

Slides containing organoid sections were dewaxed through xylene (Fisher Scientific, Loughborough, UK) submersion for 5 min and rehydrated through submersion in ethanol at 100%, 90% and 70% for 5 min. Antigen retrieval was performed by submerging slides in boiling 10 mm sodium citrate buffer (Merck), before washing with PBS. Samples were then blocked through the addition of Goat serum (Zytochem Plus, 2bscientific, Kirtlington, UK) for 1 h. Primary antibodies diluted in PBS were added for 1 h, and secondary antibodies were incubated for 1 h in the dark. Coverslips were mounted using VECTASHIELD Vibrance [TM] Antifade Mounting Medium with DAPI (2bscientific, Kirtlington, UK) for imaging. Slides were visualised by the Leica DM4000 system. Organoid staining was scored on a scale of 1–5 by an independent blinded researcher. The following antibodies were used: OLFM4 (1:200; Catalogue number: 39141, Cell Signalling), Alexa Flour Goat anti‐rabbit AF568 (1:50; Catalogue number: A‐11011, Abcam) and B‐catenin (1:100; Catalogue number: 610154, BD Biosciences, CA, USA).

### 2.14 RNA‐seq

Samples for RNA‐seq analysis were prepared by culturing cells in standard media conditions with overexpression of the CHRDL2 gene through doxycycline‐inducible expression. Doxycycline was given in quantities of 10 μg·mL^−1^, 1 μg ·mL^−1^ and 0.1 μg ·mL^−1^ or DMSO control as described previously.

RNA‐seq was performed by the Oxford Genomic centre. Data for bioinformatics analysis were given in the format of fastq raw reads. Data were analysed using the open‐source software package Tuxedo Suite. tophat2 and bowtie2 were used to map paired‐end reads to the reference Homo sapiens genome build GRCh38. GENCODE38 was used as the reference human genome annotation.

Aligned reads were filtered for quality using Samtools with the minimum selection threshold of 30. Transcript assembly and quantification were done through Cufflinks, and differential expression analysis was achieved through the use of Cuffdiff software. Differential expression was expressed in the form of log_2_ fold change between sample and control.

### 2.15 Data visualisation and R

Data were cleaned, and significant data were extracted using R software. Graphs were generated using R studio 4.1.0 using libraries ggplot2 and heatmap2.

Gene‐set‐enrichment analysis was performed using the GSEA software 4.2.3. The Chip annotation platform used was Human_Ensembl_Transcript_ID_MSigDB.v7.5.1.chip.

Gene sets used:

- c6.all.v7.5.1.symbols.gmt
- h.all.v7.5.1.symbols.gmt
- GOBP_REGULATION_OF_BMP_SIGNALING_PATHWAY
- enplot_REACTOME_PI3K_AKT_SIGNALING_IN_CANCER_13
- enplot_GOMF_BMP_RECEPTOR_BINDING_58
- WP_NRF2_PATHWAY.v2023.1.Hs.

## 3 Results

### 3.1 CHRDL2 overexpression reduces cellular proliferation and enhances migration through WNT activation

**Fig. 1:**
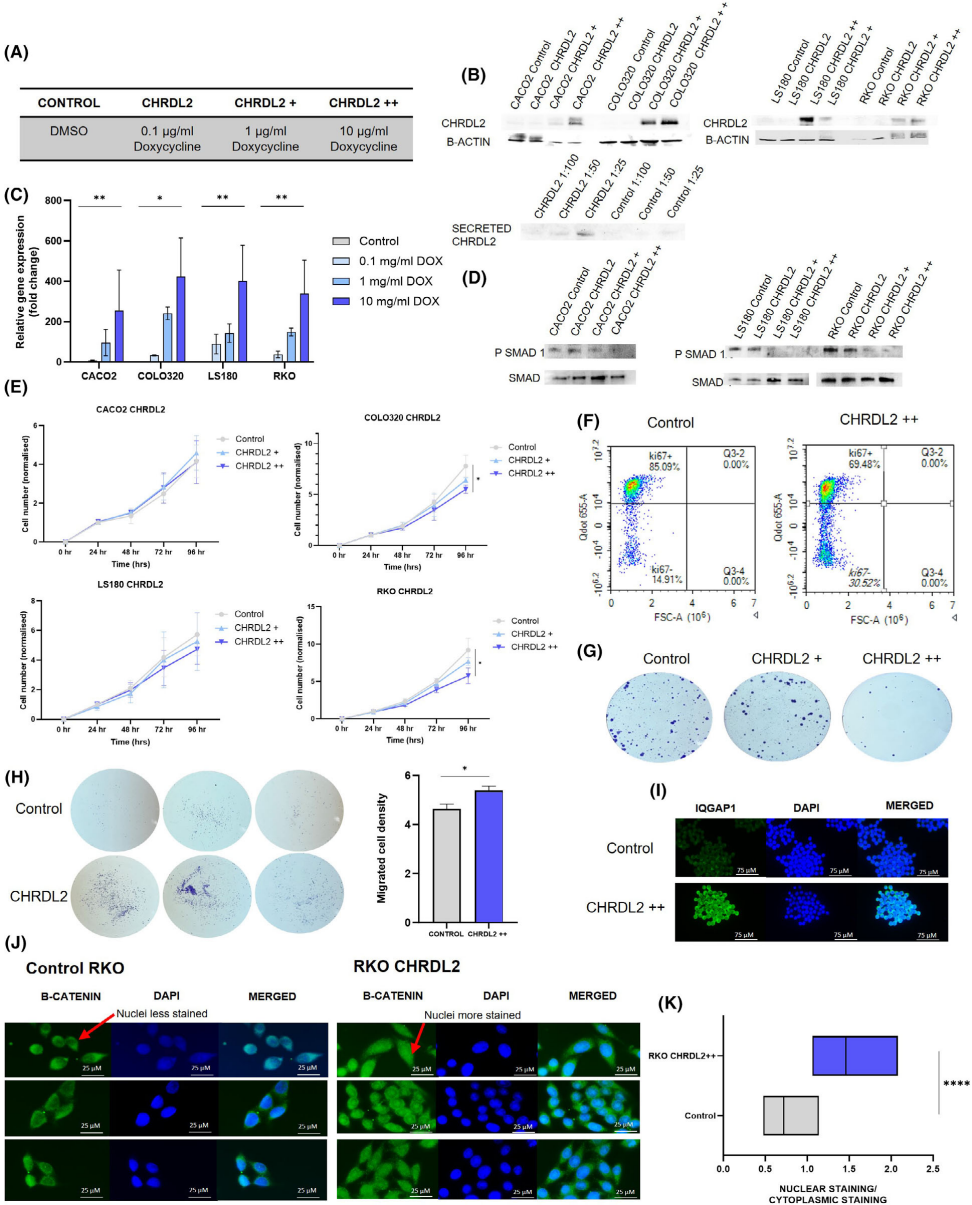
Inducible CHRDL2 overexpression in colorectal cancer (CRC) cell lines alters proliferation and migration. (A) Table of doxycycline treatment used: briefly control cells were treated with dimethyl sulfoxide (DMSO), test cells were treated with doxycycline at: 0.1 μg·mL^−1^ (CHRDL2), 1 μg·mL^−1^ (CHRDL2+) or 10 μg·mL^−1^ (CHRDL2++) to induce expression from the CHRDL2 transgene. (B) Western blotting of corresponding protein levels of CHRDL2 in cell lines with lentiviral overexpression, and secreted CHRDL2 present in cell culture media. Representative image, *N* = 3. (C) qPCR of mRNA levels of CHRDL2 expressed as fold change in 4 experimental cell lines. Cell lines were grown with doxycycline at: 0.1, 1 or 10 μg·mL^−1^ to induce expression. *T*‐test; RKO DMSO‐10 μg·mL^−1^ *P* < 0.01, COLO320 DMSO‐10 μg·mL^−1^ *P* < 0.05, CACO2 DMSO‐10 μg·mL^−1^ *P* < 0.01, LS180 DMSO‐10 μg·mL^−1^ *P* < 0.01. *N* = 3, *t*‐test. (D) Western blotting of SMAD1/5 phosphorylation in cell lines overexpressing CHRDL2. Representative image, *N* = 3. (E) MTT ((3‐(4,5‐dimethylthiazol‐2‐yl)‐2,5‐diphenyltetrazolium bromide)) assay of cellular proliferation of CHRDL2 cell lines. In COLO320 cells, two‐way ANOVA between Control and CHRDL2++ was *P* < 0.0118. LS180 cells two‐way (analysis of variance) ANOVA between Control and CHRDL2 = *P* < 0.0107. Control and CHRDL2++ *P* < 0.0114. RKO cells Control and CHRDL2++ *P* < 0.0181. One‐way ANOVA at 96 h was also performed: CACO2 *P* < 0.44, COLO320 *P* < 0.0411, LS180 *P* < 0.121, RKO *P* < 0.0476. *N* = 3. (F) Flow cytometry analysis of COLO320 cells given CHRDL2++ overexpression using Ki67 antibody staining as a marker of proliferating cells. Proportion of Ki67 cells decreased with CHRDL2++ overexpression. Representative image *N* = 3. (G) Crystal violet staining of colonies of RKO cells treated with 1 μg·mL^−1^ and 10 μg·mL^−1^ doxycycline to induce CHRDL2 expression. Representative image *N* = 3. (H) Crystal violet staining of migrated COLO320 cells with 10 μg·mL^−1^ doxycycline to induce CHRDL2 expression. Quantification on ImageJ shows significant increase in number of migrating cells with CHRD2++, *t*‐test *P* < 0.0449. *N* = 3. (I) Immunofluorescence staining of IQGAP1 on COLO320 cells with 10 μg·mL^−1^ doxycycline to induce CHRDL2 expression. *N* = 3. (J) Immunofluorescence staining B‐catenin on RKO cells with 10 μg·mL^−1^ doxycycline to induce CHRDL2 expression. *N* = 3. (K) Quantification of B‐catenin nuclear staining over cytoplasmic staining on RKO cells with 10 μg·mL^−1^ doxycycline to induce CHRDL2 expression *N* = 3. *T*‐test *P* < 0.0001. In all panels *P < 0.05, **P < 0.01, ****P < 0.0001, ns, *P* > 0.05. Error bars given as ± SEM. Scale bar indicates 25 μm.

To determine the effects of CHRDL2 on colorectal cancer cells, we transduced four extensively characterised CRC cell lines with a virally packaged doxycycline‐inducible overexpression system for full‐length *CHRDL2* cDNA. Colorectal adenocarcinoma cell lines were deliberately chosen to encompass a range of *CHRDL2* and BMP expression levels, as well as genetic mutations: CACO2 and LS180 (moderate CHRDL2), COLO320 and RKO (very low) (Fig. S1A–C). Doxycycline was given in 3 concentrations to cell lines: 0.1 μg·mL^−1^ (CHRDL2), 1 μg·mL^−1^ (CHRDL2+) or 10 μg·mL^−1^ (CHRDL2++) (Fig. 1A) to induce expression. qPCR and western blotting confirmed overexpression of CHRDL2 at the RNA and protein levels, respectively (Fig. 1B,C, Fig. S1D). BMP antagonism was shown through assessing levels of phosphorylated SMAD 1/5 (Fig. 1D, Fig. S1E). Bands were normalised to total SMAD1 levels. Conditioned media from CHRDL2‐overexpressing cell lines was also collected, and secreted CHRDL2 protein was found to be present in the media (Fig. 1B, Fig. S1F).

Increased proliferation is a hallmark of cancer cells. Therefore, we measured the effects of CHRDL2 overexpression on cellular proliferation in our cell lines. As seen in Fig. 1E, cell growth was slightly reduced during overexpression of CHRDL2 in LS180 and RKO (*P* < 0.0114, *P* < 0.0181). As seen in Fig. 1F, CHRDL2 overexpression decreased the proportion of proliferating cells, marked by Ki67+ (Fig. S2A, *P* < 0.05). This is further supported by a reduction in Ki67 immunofluorescence staining (*P* < 0.005) (Fig. S2B,C). In Ki67+ populations, CHRDL2 increased the number of S phase cells and lowered the proportion in G2 phase, possibly reflecting a decreased rate of proliferation (Fig. S2D). Investigation of the colony forming competency (clonogenicity) of CHRDL2 overexpressing cells revealed that clonogenic potential was reduced (Fig. 1G). This was found in all four tested cell lines (Fig. S2E, *P* < 0.01). Overall, contrary to our hypothesis, CHRDL2 overexpression appears to reduce proliferation and colony formation.

Migratory ability is a further measure of stem‐cell competency, so to measure this in cells with CHRDL2 overexpression, COLO320 cells were seeded in transwell inserts. The number of cells migrating through a porous membrane to the lower chamber was quantified. As seen in Fig. 1H, CHRDL2 overexpression significantly increased the number of migrated cells (*P* < 0.0449), suggesting increased migratory ability, a hallmark of cancer stem cells. This is supported by an increase in the expression of IQGAP1 *P* < 0.0005 (Fig. 1I, Fig. S2F), a marker of adherence which is associated with the metastatic competency of cancer cells [31].

Next, we investigated whether CHRDL2 expression increased the propensity of epithelial cells to gain these stem‐like qualities through upregulation of WNT signalling through BMP inhibition. Increased WNT signalling was shown by enhancement of B‐catenin staining in the nuclei over cytoplasmic staining. As seen in Fig. 1J,K, CHRDL2 overexpression increased B‐catenin nuclear localisation, a hallmark of WNT signalling (*P* < 0.005, Fig. 1K), confirming the hypothesis that CHRDL2 increases WNT signalling through BMP inhibition.

These data, along with the reduction in SMAD phosphorylation, confirm CHRDL2 as a BMP antagonist and therefore increases cellular WNT signalling. This appears to result in enhanced migratory ability, however, also supports a slower‐growing cellular phenotype.

### 3.2 CHRDL2 increases resistance to common chemotherapy

Another characteristic of cancer stem cells is resistance to chemotherapy [32]. In light of this, our study aimed to evaluate the response of our experimental cell lines to the three most common chemotherapy agents employed in the treatment of CRC.

**Fig. 2:**
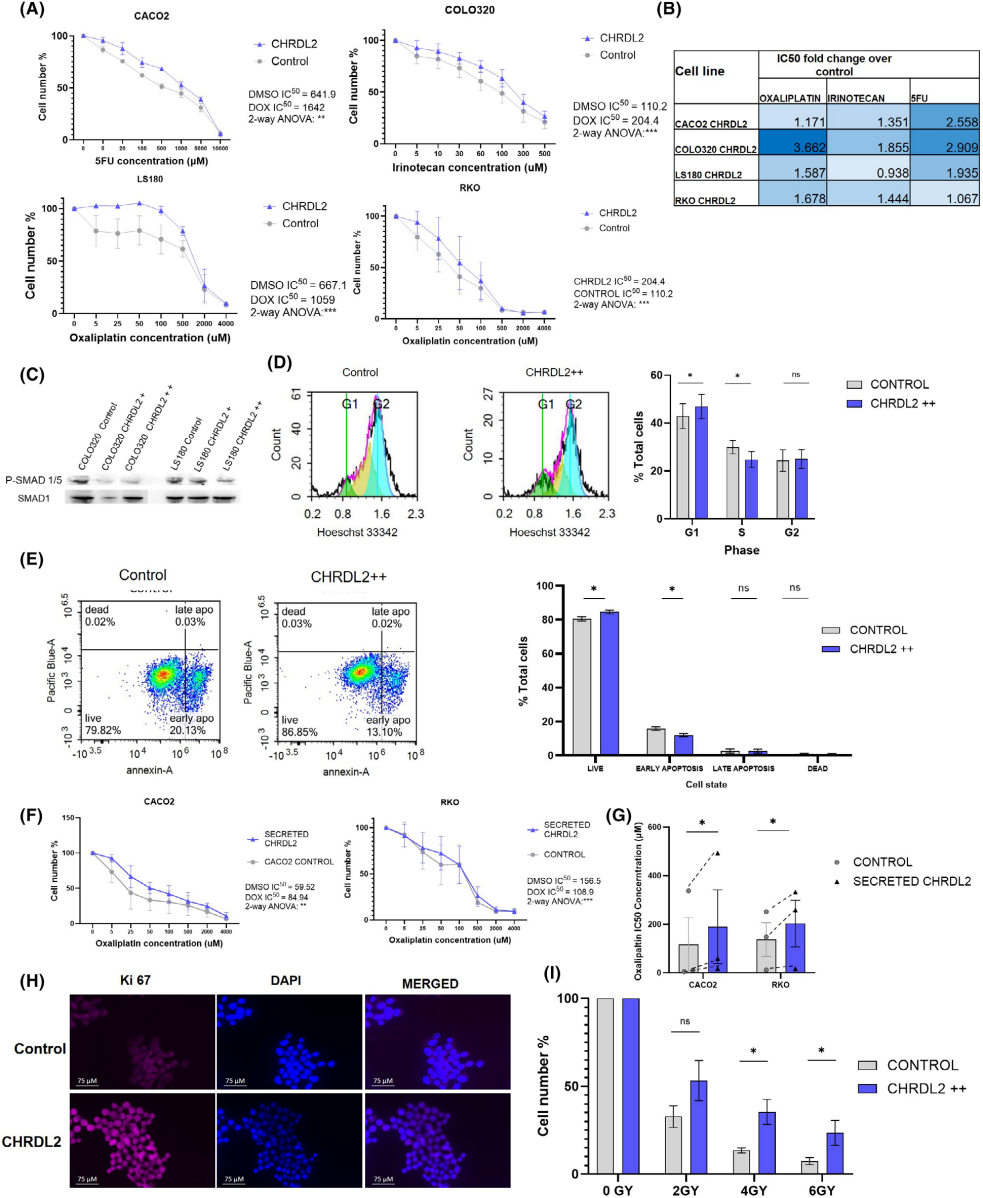
CHRDL2 overexpression increases resistance to common colorectal cancer (CRC) chemotherapies. (A) Drug dose–response curves using CACO2 cells and 5‐fluorouracil (5FU), COLO320 cells and Irinotecan, and LS180 and RKO cells and oxaliplatin *N* = 3. Two‐way (analysis of variance) ANOVA was used to find differences between curves, *P* < 0.0068, *P* < 0.0001, *P* < 0.0006, *P* < 0.005, respectively. (B) Table of ratio differences in IC50 values between cells treated with 10 μg·mL^−1^ doxycycline (CHRDL2++) and control for each chemotherapy drug and cell line. *N* = 3. (C) Western blotting of SMAD1/5 phosphorylation in cell lines overexpressing CHRDL2 through addition of doxycycline at 1 μg·mL^−1^ (CHRDL2+) or 10 μg·mL^−1^ (CHRDL2++) and treated with 10 μm 5FU. Representative image *N* = 3. (D) Flow cytometry analysis of COLO320 cells treated with oxaliplatin and CHRDL2 overexpression. CHRDL2 increased the number of cells in G1 phase. (*t*‐test *P* < 0.003), and decreased cells in S phase (*t*‐test, *P* < 0.05) *N* = 3. (E) Flow cytometry analysis of COLO320 cells treated with oxaliplatin and CHRDL2 overexpression stained with Annexin‐5 as a marker of apoptosis. Quantification of cell percentages of live, apoptotic and dead cells in COLO320 cells treated with oxaliplatin and given CHRDL2 overexpression. CHRDL2 increased the % of live cells (*t*‐test *P* < 0.03) and decreased the % of early apoptotic cells (*P* < 0.023). *N* = 3. (F) Drug dose–response curves of CACO2 and RKO cells with CHRDL2 conditioned media and oxaliplatin *N* = 3. Two‐way ANOVA was used to find differences between curves, *P* < 0.005, *P* < 0.0001. (G) Average IC50 values for chemotherapy drug oxaliplatin, on cell lines CACO2 and RKO with CHRDL2 conditioned media. Conditioned media was harvested from corresponding cell lines and administered to parental cell lines to mimic CHRDL2 paracrine signalling at the same level of the doxycycline‐inducible expression system. CHRDL2 against control *t*‐test *P* < 0.0305. *N* = 3. (H) Immunofluorescence staining of Ki67 on COLO320 cells treated with 5 μm oxaliplatin. (I) Cell count after irradiation of RKO cells overexpressing CHRDL2++ *N* = 3. *T*‐test 4GY: *P* < 0.038, 6GY: *P* < 0.0241. In all panels *P < 0.05, ns, *P* > 0.05. Error bars given as ± SEM. Scale bar indicates 25 μm.

We treated CHRDL2++ cells with chemotherapy drugs, 5‐Fluorouracil (5FU), irinotecan and oxaliplatin and assessed cellular response via MTS assay. Figure 2A shows the reduction in cell number with increasing chemotherapy concentration (μm). Control cell lines were plotted together with cell lines with CHRDL2 overexpression, and the half maximal inhibitory concentration (IC50) values were calculated (Fig. 2A,B; Fig. S3). CHRDL2 overexpression significantly increased resistance to chemotherapy in all cell lines (*P* < 0.01) as shown by elevated IC50 values (Fig. S3). Average increases in IC50 values during CHRDL2 overexpression for each drug and cell line can be observed in Fig. 2B. The greatest increase in survival (exhibited by ratios of IC50s) was seen in COLO320 cells treated with oxaliplatin, which had a 3.6‐fold increase. Cells treated with the IC50 value of 5FU had decreased expression of P‐SMAD1/(Fig. 2C), compared with untreated CHRDL2 overexpressing cells (Fig. 1D). This indicates that the cells with the highest CHRDL2 expression were able to survive the chemotherapy (Fig. 2C, Fig. S4A).

Flow cytometry of COLO320 cells treated with oxaliplatin (Fig. 2D) showed a clear increase in the number of cells in S phase (in both control and CHRDL2++ cells) compared to the untreated cells (Fig. S2D). This is probably due to stalling of replication forks due to DNA damage exerted by chemotherapy and activation of the S/G2 checkpoint. Interestingly, cells with CHRDL2 overexpression displayed a smaller increase in cells stalled in S Phase compared to controls and therefore showed a greater proportion present in G1 phase. This is the opposite of that of untreated cells, where CHRDL2 increased the number of cells in S Phase possible due to slower cell division. Further flow cytometry analysis following chemotherapy treatment revealed CHRDL2 overexpression decreased the number of cells that had entered early apoptosis (*P* < 0.05) (Fig. 2E) demonstrating that CHRDL2 overexpressing cells have the ability to evade apoptosis.

Secreted CHRDL2 from conditioned media was also used on our parental non‐CHRDL2 expressing cell lines to assess paracrine signalling. Conditioned media was harvested from corresponding cell lines with the inducible CHRDL2 transgene and the parental control cells. Induction of CHRDL2 to generate conditioned media was carried out using the same concentration and duration of doxycycline treatment as the cells in Fig. 2A. Again, secreted CHRDL2 increases cellular survival during chemotherapy in the same manner as our intracellular CHRDL2 overexpression system, *P* < 0.005 with elevated IC50 values (Fig. 2F,G).

To further analyse the survival capabilities of CHRDL2 overexpressing cells during chemotherapy, Ki67 staining was performed. As seen in Fig. 2H, CHRDL2 overexpression significantly increased the number of proliferating cells during treatment with a low dose (IC25) of chemotherapy drug oxaliplatin. This is supported by flow cytometry analysis (*P* < 0.0055, Fig. S4B) and an upregulation in Ki67 (*P* < 0.0064, Fig. S4C).

Resistance to irradiation, along with chemotherapy resistance, is also attributed to more aggressive cancers that evade treatment. ICSs have been shown to have increased resistance to irradiation; therefore, we sought to measure the effects of CHRDL2 overexpression on cell survival during X‐ray irradiation. Cells were treated with 0 GY, 2 GY, 4 GY or 6 GY X‐ray irradiation, and cell viability was assessed. As seen in Fig. 2I, CHRDL2 overexpression increased cell survival at 4GY and 6GY radiation (*P* < 0.03, *P* < 0.02).

### 3.3 CHRDL2 overexpression decreases DNA damage during chemotherapy treatment

Next, we investigated the mechanism through which CHRDL2 promotes cell survival during chemotherapy treatment. The chemotherapy agent oxaliplatin is known to cause DNA intra‐strand cross‐linking, resulting in double‐strand breaks (DSBs), cell‐cycle arrest and apoptosis [33]. Therefore, quantification of DSBs in cells treated with oxaliplatin was performed on COLO320 CHRDL2++ cells using immunofluorescence staining of γH2AX and Ku70. Quantification of DNA repair proteins ATM and RAD21 was also performed to assess whether CHRDL2 protects cells from DNA damage through upregulation of DNA repair pathways.

**Fig. 3:**
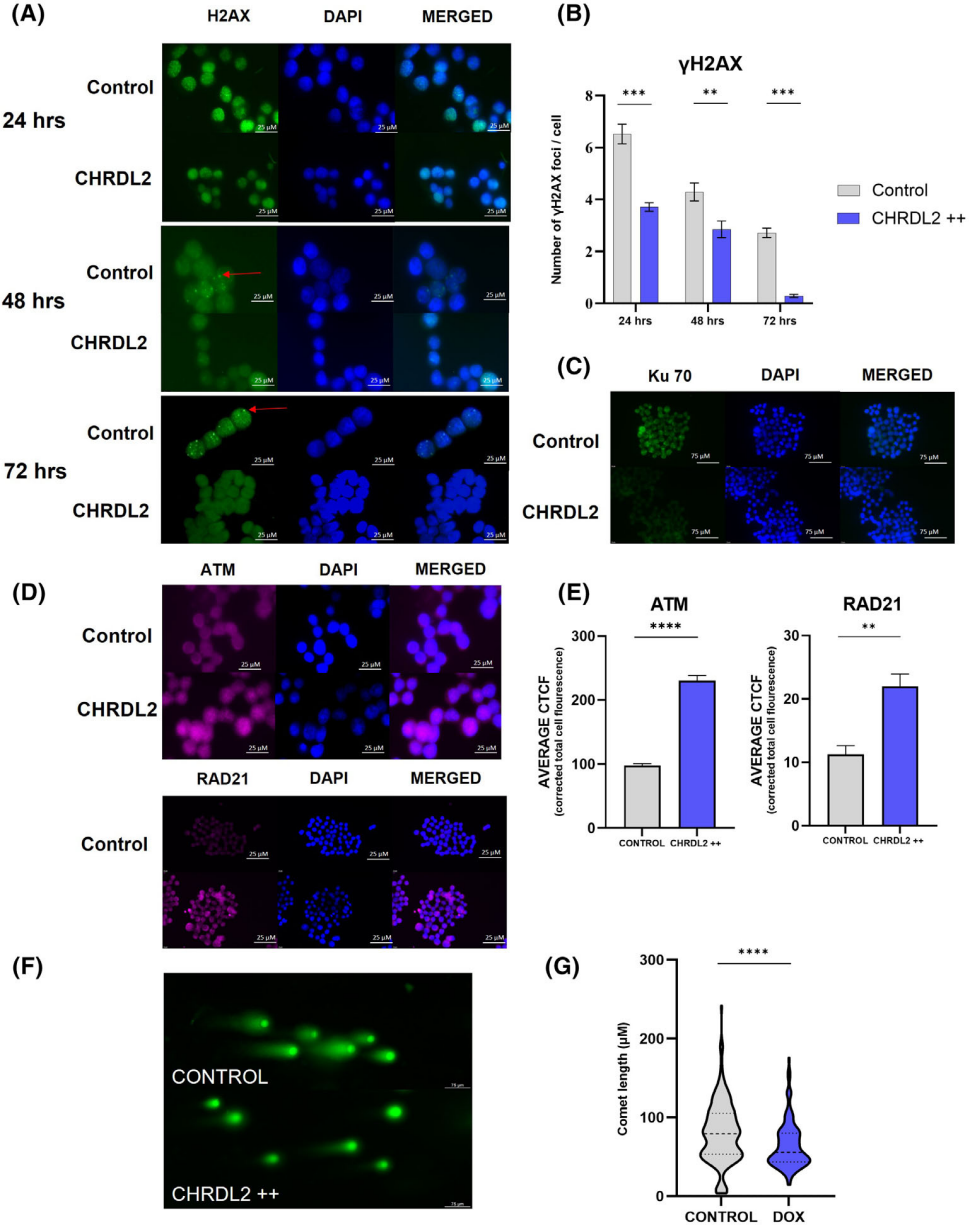
CHRDL2 overexpression decreases DNA damage during chemotherapy treatment and enhances expression of DNA repair pathways. (A) Representative image of immunofluorescence of γH2AX on COLO320 cells treated with 5 μm oxaliplatin at 24, 48 and 72 h. Foci indicated by red arrows *N* = 3. (B) Quantification of γH2AX foci in COLO320 cells overexpressing CHRDL2 treated with 5 μm oxaliplatin at 24, 48 and 72 h. Cells were treated with (dimethyl sulfoxide) DMSO control reagent, or 10 μg·mL^−1^ Doxycycline to induce CHRDL2 (CHRDL2++) overexpression. *T*‐test; 24 h *P* < 0.0001, 48 h *P* < 0.01, 72 h *P* < 0.0001. *N* = 3. (C) Immunofluorescence staining of Ku 70 on COLO320 cells treated with 5 μm oxaliplatin. Representative images *N* = 3. (D) Immunofluorescence staining of ATM and RAD21 on COLO320 cells treated with 5 μm oxaliplatin. Representative images *N* = 3. (E) Quantification of ATM and RAD21 staining on COLO320 cells. Immunofluorescence given as Corrected Total Cell Fluorescence (CTCF). Cells were treated with DMSO control reagent, or doxycycline to induce CHRDL2++ overexpression. *T*‐test; *P* < 0.0001 and *P* < 0.0023, respectively. *N* = 3. (F) Comet assay of RKO cells treated with IC50 Oxaliplatin. Cells were then treated with CHRDL2 ++ overexpression or a control representative image *N* = 3. (G) Quantification of Comet assay, *t*‐test *P* < 0.0001. *N* = 3. In all panels **P < 0.01, ***P < 0.001, ****P < 0.0001, ns, *P* > 0.05. Error bars given as ± SEM. Quantification carried out using ImageJ. Scale bar indicates 25 μm in panels A, C and D. Scale bar indicates 75 μm in panel F.

Figure 3A images show DSBs in cells treated with a low dose of oxaliplatin (approx. IC25) after 24‐, 48‐ and 72‐h posttreatment. Quantification using ImageJ demonstrated that CHRDL2 overexpressing cells had significantly fewer γH2AX foci compared to the control at each time point (Fig. 3B *P* < 0.01, *t*‐test). This difference was notably increased at 72 h compared to 24 h. This suggests that CHRDL2 does not necessarily protect cells from DNA damage but rather acts to accelerate the repair of DNA damage when compared to control cells. Similarly, after 72 h there was a decrease in the presence of Ku70, which binds to DSBs to facilitate nonhomologous end‐joining (NHEJ), in COLO320 CHRDL2 cells (*P* < 0.0057, Fig. 3C and Fig. S4D). This further confirms a reduction in DNA damage through CHRDL2 upregulation.

This is supported by Fig. 3D and E, in which we observed significantly (*P* < 0.0001) increased ATM and RAD21 (*P* < 0.0023) in CHRDL2 overexpressing cells compared to the control, suggesting upregulation of DSB damage response pathways in which ATM serves as a master transducer. Furthermore, we have shown increased expression of ERCC1 and PCNA, which serve in the repair of DNA crosslinks and single‐strand breaks (SSBs), as well as ARTEMIS, which is involved in DSB repair through NHEJ (Fig. S5). This suggests a global upregulation of DNA repair pathways by CHRDL2 overexpression following chemotherapy treatment.

DSB marker γH2AX is also known to accumulate during cellular senescence. However, since we found no difference in P53 expression (a marker of senescence, Fig. S5) in our CHRDL2 overexpressing cells, it is likely that upregulation of DNA damage pathways in CHRDL2++ cells protects against DNA damage by chemotherapy.

We have further demonstrated the ability of CHRDL2 overexpression to reduce DNA damage during chemotherapy by alkaline comet assay, as observed in Fig. 3F and G. Cells were treated in the same manner with IC25 oxaliplatin. We observed cells with CHRDL2 overexpression had shorter ‘tails’ to their comets, showing less fragmented or damaged DNA. Quantification using ImageJ confirmed this, with CHRDL2++ cells having significantly decreased tail length compared to control cells (*P* < 0.0001).

Here, we have shown that not only does CHRDL2 overexpression reduce DNA damage during chemotherapy, but it also results in activated DNA repair pathways, clearing DNA damage. This allows our cancer cell models to increase their survival capabilities during chemo‐ and radiotherapy.

### 3.4 CHRDL2 decreases organoid budding and increases stem‐cell markers

CRC cell lines exhibit a multitude of abnormalities, characterised by numerous mutations, heightened WNT signalling, and impaired DNA repair mechanisms. Consequently, we also explored the impact of CHRDL2 overexpression on normal ISCs within an organoid model, aiming to shed light on the role of CHRDL2 in tumour initiation.

To replicate the stem‐cell niche and overexpression of CHRDL2 paracrine signalling, which typically originates from mesenchymal cells, we established murine intestinal organoids, providing a three‐dimensional platform for modelling the effects of CHRDL2. These organoids were exposed to secreted forms of CHRDL2 in the form of conditioned media obtained from our cell lines. As a control, conditioned media from parental (non‐CHRDL2 expressing) cells undergoing doxycycline treatment was also collected.

Small intestinal organoids showcase distinct morphological features, characterised by a villi‐bud‐like configuration, with the outer epithelial layer forming distinct protrusions and invaginations. Prior investigations have examined the gene expression profiles of intestinal organoids, revealing that epithelial cells within the ‘buds’ of the organoids exhibit crypt‐like expression patterns, while the evaginations display villus‐like expression [34].

**Fig. 4:**
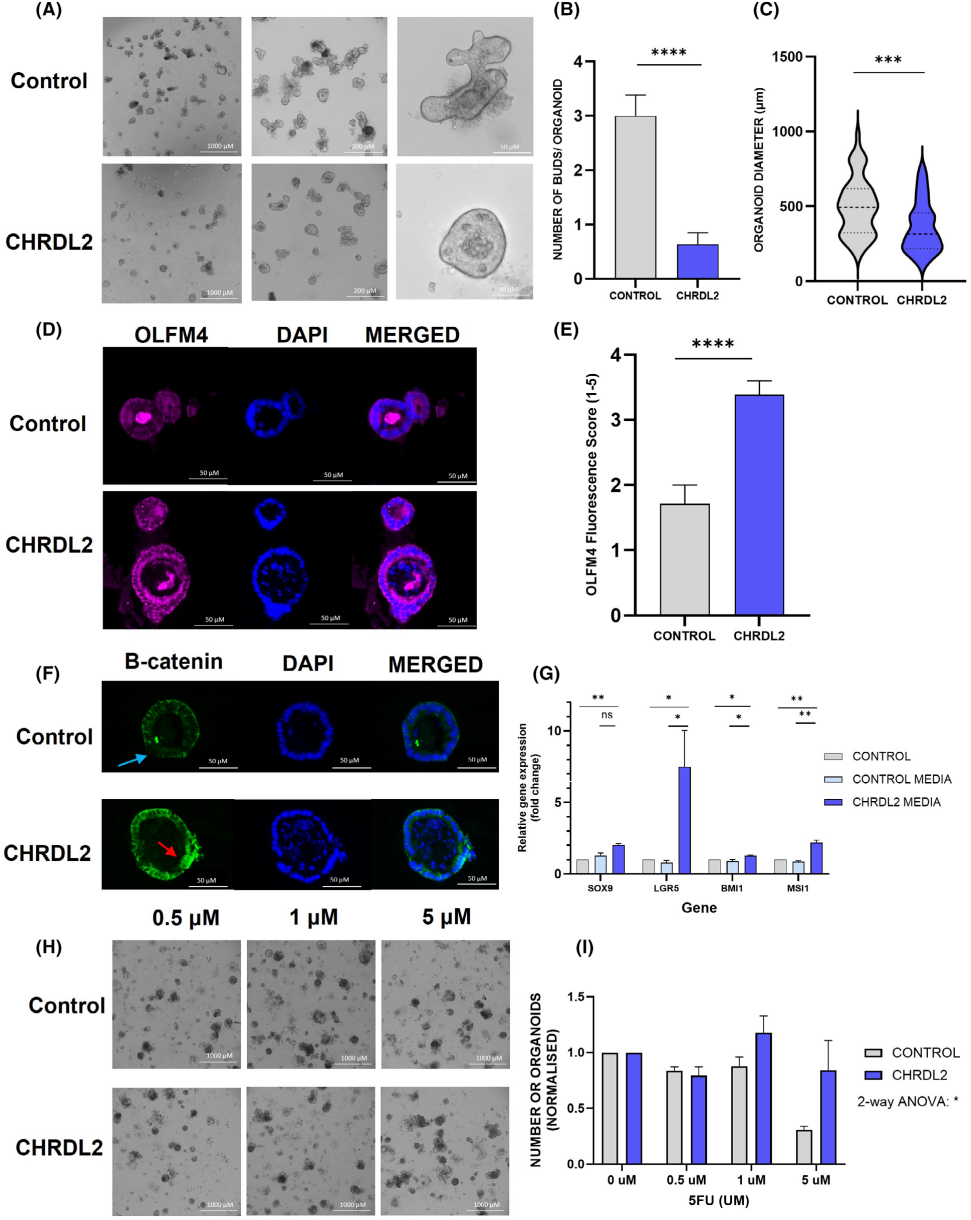
Secreted CHRDL2 decreases murine small intestinal organoid differentiation and increases stem‐cell marker expression. (A) Image of murine‐derived organoids treated with conditioned media containing secreted forms of CHRDL2 compared to conditioned media from control cells with no CHRDL2 overexpression representative images *N* = 3. (B) Quantification of buds per organoid in CHRDL2‐treated murine organoids compared to a control. *T*‐test *P* < 0.0001 *N* = 3. (C) Quantification of average organoid diameter in CHRDL2‐treated murine organoids compared to a control. *T*‐test *P* < 0.001 *N* = 3. (D) Immunofluorescence staining of OLFM4 protein on murine organoids treated with secreted CHRDL2 compared to a control after 1‐week representative images *N* = 3. (E) Quantification of immunofluorescence scoring of OLFM4 on murine organoids treated with secreted CHRDL2 compared to a control. *T*‐test *P* < 0.0001 *N* = 3. (F) Immunofluorescent staining of β‐catenin localisation in murine small intestinal organoids upon CHRDL2 addition and a control Representative images *N* = 3. (G) qPCR of stem‐cell markers from CHRDL2‐treated murine organoids compared to a control. *T*‐test SOX9 *P* < 0.0014, LGR5 *P* < 0.04, *P* < 0.043, BMI1, *P* < 0.49, *P* < 0.0113, MSI1 *P* < 0.0067, *P* < 0.009. *N* = 3. (H) Images of murine‐derived organoids treated with conditioned media containing secreted forms of CHRDL2 compared to a control and 5‐fluorouracil (5FU). Image taken 96‐h posttreatment representative images *N* = 3. (I) Quantification of number of live organoids in CHRDL2‐treated organoids compared to a control. *N* = 3. Two‐way ANOVA comparing the number of live organoids in control and CHRDL2‐treated groups at all drug concentrations, *P* < 0.0442. In all panels *P < 0.05, **P < 0.01, ***P < 0.001, ****P < 0.0001, ns, *P* > 0.05. Error bars given as ± SEM. Scale bar indicates 1000, 200, 50 and 40 μm in panel A. Scale bar indicates 50 μm in panel D and F. Scale bar indicates 1000 μm in panel H.

As seen in Fig. 4A, upon the addition of extrinsic CHRDL2 to organoids, a noticeable reduction in the number of differentiated buds was observed (*P* < 0.001) (Fig. 4A,B). Organoids developed smaller and more circular characteristics, suggesting slower growth similar to our observations in cell lines (*P* < 0.001) (Fig. 4A,C). This is supported by the presence of olfactomedin‐4 (OLFM4), a marker for LGR5+ stem cells and downstream target of WNT signalling [35], which was increased in CHRDL2‐treated organoids (*P* < 0.0010) (Fig. 4D,E). As seen in Fig. 4F, in control organoids, β‐catenin is localised to the outer cellular membrane (blue arrow), whereas upon extrinsic CHRDL2 treatment, β‐catenin can be observed in the cytoplasm and nucleus of organoid cells (red arrow). Moreover, as illustrated in Fig. 4G, we observed a significant increase compared to the controls (*P* < 0.05) in the expression of stem cell markers LGR5 (indicating crypt CBCs) and BMI1 (slow‐cycling crypt stem cells), and also SOX9 and MSI1. Furthermore, we have shown an enhancement of WNT signalling through the observed increase in β‐catenin nuclear localisation.

CHRDL2‐treated organoids exhibited resistance to chemotherapy treatment, as observed in Fig. 4H,I. At 5 μm 5FU, control organoid numbers diminished to less than half of untreated samples whereas CHRDL2‐treated organoids showed little to no death. Taken with the increased stem‐cell markers, these findings collectively suggest that exposing intestinal organoids to CHRDL2 diminishes differentiation through increased WNT signalling and enhances stem‐cell numbers, leading to increased chemotherapy resistance.

### 3.5 CHRDL2 enhances cancer stem‐cell pathways

**Fig. 5:**
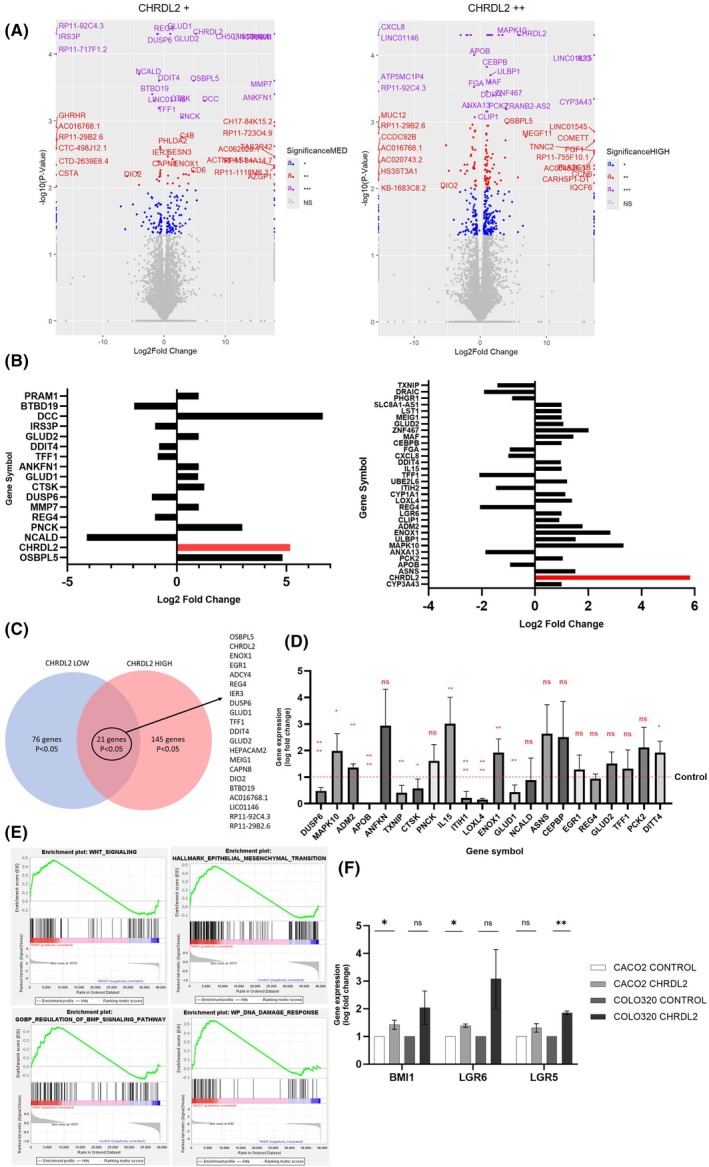
RNA‐seq analysis demonstrates that CHRDL2 expression enhances cancer stem cell and other cancer hallmark pathways. RNA‐seq analysis of CACO2 cells treated with 1 μg·mL^−1^ (CHRDL2+) or 10 μg·mL^−1^ (CHRDL2++) compared to control (dimethyl sulfoxide) DMSO‐treated cells *N* = 3. (A) Volcano plot of differentially expressed genes from cells with CHRDL2 overexpression (CHRDL2+ and CHRDL2++) from RNA‐seq analysis. Expressed as Log_2_ Fold change against control. (B) Bar plot of significantly differentially expressed genes in CHRDL2+ and CHRDL2++ cells. Genes included pass the threshold of *P* < 0.001 for CHRDL2+ and *P* < 0.001 for CHRDL2++. (C) Intersect of highly differentially expressed genes in both the CHRDL2+ and CHRDL2++ treated groups *P* < 0.05. 21 genes were differentially expressed in both groups. (D) qPCR validation of differentially expressed genes from RNA‐seq data in CACO2 and COLO320 cells *N* = 3. (E) GSEA plots of differentially expressed pathways in CACO2 cells treated with CHRDL2++. (F) qPCR validation of stem‐cell markers in CACO2 and COLO320 cells *N* = 3. *T*‐test; CACO2 BMI1 *P* < 0.05, CACO2 LGR6 *P* < 0.05, COLO320 LGR5 *P* < 0.01. *N* = 3. In all panels *P < 0.05, **P < 0.01, ns, *P* > 0.05. Error bars given as ± SEM.

To elucidate pathways in which CHRDL2 overexpression acts, RNA‐seq analysis on CACO2 cells given CHRDL2+ and CHRDL2++ treatment was performed with DMSO‐treated cells as a baseline. Differential expression analysis was carried out on RNA‐seq data (Fig. 5A). 76 and 145 differentially expressed genes were identified in the CHRDL2 + and CHRDL2++ groups, respectively, at *P* < 0.05 (Fig. 5B). From this, we selected the 22 genes that were differentially expressed in both CHRDL2+ and CHRDL2++ vs control cells for downstream analysis (Fig. 5C). qPCR was used to confirm expression changes in biological replicates in both CACO2 and COLO320 cells (Fig. 5D).

Gene‐set‐enrichment analysis (GSEA) on the entire RNA‐seq dataset revealed upregulation of the hallmark WNT signalling pathway (*P* < 0.001) and enrichment of genes that modulate the frequency, rate or extent of the activity of the BMP receptor signalling pathway (*P* < 0.05). Furthermore, BMP‐specific target gene ID1, which is induced upon BMP stimulation, was downregulated in our data set by CHRDL2 overexpression. APOA4, APOB and APOC3, which have been shown to be induced specifically by BMP 2 and 4, were also reduced by CHRDL2 overexpression [36]. This suggests that CHRDL2 overexpression results in the direct reduction of BMP signalling mediated by BMP 2 and 4. This increase in WNT signalling and modulation of BMP signalling (Fig. 5E) verifies CHRDL2's role as a BMP antagonist in colon cancer cells. The MYC signalling pathway and LEF1 signalling, which are downstream transducers of WNT signalling, were also upregulated.

We further investigated stem‐cell markers *LGR5* and *LGR6*, as well as B lymphoma Mo‐MLV insertion region 1 homologue (*BMI1*) and showed upregulation by QPCR, with *LGR6*, a WNT transducer, also upregulated in RNA‐seq data in the CHRDL2++ treatment (Fig. 5F). We noted that BMI1 pathways were also highlighted by GSEA (Fig. S6) a stem‐cell defining pathway and correlating with our Q‐PCR data.

GSEA revealed upregulation of the cancer hallmark pathways, epithelial to mesenchymal transition (EMT) (*P* < 0.001), and angiogenesis, which are frequently upregulated in metastatic colorectal cancers (Fig. S6). DNA repair pathways were also significantly upregulated including key DSB repair genes BRCA1, RAD51 and RAD52, supporting our findings with respect to chemo/radiotherapy resistance (*P* < 0.05). There was also significant upregulation of RAF and MTOR signalling, which are often modulated during cancer progression. Furthermore, cell cycle‐related genes upregulated by Rb knockout were also upregulated by CHRDL2, suggesting an increase in cell‐cycle protein signalling (Fig. S6). Gene ontology analysis supports these findings with enrichment in biological processes such as cellular adhesion, apoptosis and differentiation (Fig. S7).

Interestingly, several genes that are involved in metabolism and oxidative stress were deregulated by CHRDL2, including TXNIP and ENOX1, which affect reactive oxygen species, ADM2 and PCK2, involved in metabolic stress, and PCK2 important for glucose‐independent metabolism. GSEA analysis also showed a deregulation in glycolysis signalling and a reduction in oxidative phosphorylation.

Genes involved in cancer cell invasion, CTSK and LOXL4, were also upregulated, enhancing our observations on EMT pathway markers. Further migration and invasion phenotypic observations could be due to stimulation of the RAS/RAF/MEK/ERK due to upregulation of both ANKFN1 and EGR1 [37]. Again, this is corroborated by GSEA analysis, which shows an enrichment for RAF signalling pathways (Fig. S7). We also found upregulation of MAPK10, which has been shown to inhibit apoptosis, which links to our observed increase in survivability. However, we also saw a downregulation of DUSP6, which activates dephosphorylated MAPK10.

There was also evidence of deregulation of several immune and inflammatory pathway markers by CHRDL2 overexpression. IL15 is involved in immune cell infiltration to tumour sites, and CEBP stimulates Inflammatory marker IL6. IL2 signalling was also upregulated in our GSEA data, suggesting an increase in immune infiltration (Fig. S6). ITH1, which has been shown to stabilise ECM during inflammation, was downregulated in our data and has also been shown to be lost in many cancer types. This would suggest an increase in immune and inflammation pathways by CHRDL2 overexpression.

There was an observed a downregulation of genes that are highly expressed in the healthy intestinal lining and mucosa, such as NCALD and TIFF1. NCALD, a calcium sensor, is found abundantly expressed in the intestinal tract, as well as TIFF1, which aids in the generation of the intestinal mucosa. This could signify a dedifferentiation of our cancer epithelial cells.

In summary, the differentially expressed genes and pathways due to CHRDL2 upregulation included stem‐cell maintenance, metabolic changes, invasion, migration and EMT processes, DNA repair and immune infiltration. These are all well characterised processes in cancer progression as well as treatment response, highlighting CHRDL2 as an important candidate for further biomarker and therapeutic target studies.

## 4 Discussion

The role of BMP signalling in cancer is well studied but often paradoxical; with BMP signalling shown to be necessary to prevent cancer‐associated WNT signalling and ISCs from exiting intestinal crypts [12, 25]. Conversely, it can also play a role in promoting tumorigenesis [16]. It is clear, however, that BMP antagonists play an important functional role in regulating BMP signalling and therefore could be a key biomarker in cancer progression [20]. In this study, we have confirmed that CHRDL2 represses BMP signalling in CRC cells, leading to elevated WNT signalling, and causes changes in cell growth, response to chemotherapy and stem‐cell characteristics.

Previously, CHRDL2 has been shown to bind to BMP2 and 9 to block BMP‐mediated SMAD1/5 phosphorylation signalling [27, 30]. Patient survival studies have shown CHRDL2 overexpression predicts poor prognosis in CRC patients, and mRNA expression is elevated in patient tumour tissues compared to a control. Variation near CHRDL2 has also been implicated as a cause of increased CRC risk in genome‐wide association (GWAS) studies [38, 39]. Knockdown studies of CHRDL2 have been shown to inhibit proliferation and migration in CRC, gastric cancer and osteosarcoma, and overexpression promotes cellular proliferation, migration and clonogenicity [27, 30]. Since other secreted BMP antagonists, Noggin and GREM1, have also been shown to enhance tumorigenesis and modulate intestinal cell stemness, we suggest that CHRDL2 acts in a similar manner and that this is via the BMP pathway [21, 23, 25].

Here we have used doxycycline‐inducible models to overexpress CHRDL2 at a variety of levels to investigate the transcriptional and behavioural effects of this gene. We have shown that as a BMP antagonist, CHRDL2 reduces the level of phosphorylated SMAD1/5/9 level, indicative of reduced BMP signalling, In addition, we have shown increased WNT signalling upon CHRDL2 addition in both cell lines and organoid models, demonstrated through localisation of β‐catenin to the nucleus.

Despite previous reportings of CHRDL2 increasing proliferation, we found a small decrease in proliferation in our CRC cell lines, which is reflected by a reduction in Ki‐67+ cells and reduced colony growth.

We also observed enhanced cell migration through a porous membrane of CHRDL2 overexpressing cells. CSCs have long been shown to harbour increased migratory potential, supporting our findings of increased stem‐like qualities during CHRDL2 overexpression. This is reflected in our RNA‐seq data, which shows enrichment for the EMT pathway, which relies on enhanced migratory and invasion properties of cancer cells.

Next, we identified increased resistance to the three most common forms of chemotherapy used to treat CRC, again reinforcing the propensity for survival during CHRDL2 overexpression in our CRC cell model. Standard of care for all but the early stages of CRC relies on the use of chemotherapy in combination with surgical procedures. 5‐Fluorouracil (5FU) is currently the cornerstone of chemotherapy treatment used to treat CRC [40, 41] and is used in combination with either Oxaliplatin, a diamino cyclohexane platinum compound that forms DNA adducts (Known as FOLFOX) or Irinotecan, a topoisomerase I inhibitor (known as FOLFIRI) [42]. In each cell line we tested, there was an increase in survival during chemotherapy treatment, regardless of the mechanism of action of the three chemotherapy agents. This was also seen in organoids treated with secreted CHRDL2, which had increased survival capabilities after 5FU exposure compared to a control. Chemotherapy resistance was confirmed through flow cytometry analysis that showed CHRDL2 overexpression reduced the number of cells that entered apoptosis and also increased the number of proliferating cells remaining after treatment. Additionally, we have shown that CHRDL2 overexpression increases cell survival during irradiation treatment, which can be used concurrently with chemotherapy in the treatment of CRC.

We observed upregulation of several key DNA repair proteins, such as RAD21, ATM, PCNA, ERCC1, ARTMEIS and BRCA1 signalling during chemotherapy of CHRDL2 overexpressing cells indicating hyperactive DNA damage response pathways. This is likely to be a factor in the accelerated clearing of DSBs as marked by the significantly faster reduction in γH2AX foci and Ku70. DNA damage repair genes were also shown to be upregulated by our RNA‐seq data, as shown by GSEA, although these cells had not been exposed to any chemotherapy, and therefore, we would expect only baseline DNA repair activity. Exactly which repair pathways are upregulated in response to CHRDL2 cells undergoing chemotherapy, and whether these are error prone or accurate, is an important question that remains to be addressed. In general, enhanced DNA damage response activation is also a hallmark of CSCs, which has been shown extensively to aid CSCs survival following conventional treatments, allowing the return of cancer in patients and worsened prognosis [43, 44, 45].

Through comprehensive RNA‐seq analysis and qPCR validation, we have shown that CHRDL2 overexpression increased WNT signalling and expression of stem‐cell markers, including LGR5, BMI1, LGR6, and SOX9 in 2D and 3D models. These data collectively suggest that CHRDL2 enhances stem‐cell capacity through increased WNT signalling and therefore intensifies a stem‐cell like phenotype.

However, CHRDL2 does not enhance proliferation and clonogenicity, suggesting that it could preferentially support cancer stem cells that are slower cycling as opposed to the hyperproliferative cancer stem cells. Within the intestinal crypt, normal stem cells are arranged in a hierarchy, with rapidly proliferating stem cells or crypt base columnar cells (CBCs) at the base of the crypt. A separate population of stem cells lies in the +4 position, which appear to cycle more slowly [4, 5]. Although the data are inconsistent, some studies propose that these slow‐cycling stem cells at the +4 position are marked by BMI1, which was upregulated in our RNA‐seq and qPCR data, raising the possibility that CHRDL2 enhances this slow‐cycling stem‐cell phenotype. Slow‐cycling CSCs have also been shown to be radiation resistant, similar to our CHRDL2 overexpressing cells [46, 47]. There is some conflicting evidence for the role of these slow‐cycling stem cells with a number of publications proposing that they are key for regeneration of the intestine after injury [5, 48]. However, more recent studies show that LGR5+ CBCs are also able to fulfil this role, or suggest that the two populations support each other to facilitate tissue repair [6].

Our organoid models treated with secreted CHRDL2 further confirmed our increased stem‐like hypothesis. When mouse organoids were treated with CHRDL2, they showed a significant reduction in differentiated bud formation, creating a smaller, spherical stem‐like phenotype compared to controls. Immunofluorescence staining of the stem‐cell marker OLFM4 showed upregulation in organoids treated with CHRDL2. Furthermore, stem‐cell markers *LGR5*, *MSI1*, *BMI1* and *SOX9* were increased following CHRDL2 treatment, showing that inhibition of extracellular BMP signalling can directly increase stemness in normal intestinal cells. In a recent study, another BMP antagonist, GREM1, is upregulated in the intestinal stroma in response to injury, resulting in reprogramming/dedifferentiation in intestinal epithelial cells to drive repair [49]. Conversely, treatment of human intestinal organoids with BMPs, despite causing no major morphological differences, resulted in reduced expression of stem‐cell markers, including OLFM4 [36]. Thus, inhibition of BMP signalling through CHRDL2 or other antagonists may indeed force cancer cells in the colon into a more stem‐like state, and while this may not increase the proliferation rate, it could increase the longevity and survival of these cells during treatment with DNA‐damaging therapies.

Through RNA‐seq analysis, we have also shown differential expression of other cancer biomarkers by CHRDL2, such as *EGR1*, *REG4* and *TFF1*, which have been shown to regulate proliferation, migration and metastasis. Furthermore, CHRDL2 was found to differentially impact several key cancer pathways, including the EMT pathway, MYC, MTOR, PI3/AKT and RAF. For example, DDIT4 is a regulator of MTOR and was upregulated by CHRDL2 and EGR1, which acts through PI3K/AKT, was downregulated by CHRDL2. Indeed, CHRDL2 has previously been shown to act via PI3K/AKT in osteosarcoma [30]. While the changes in BMP and WNT signalling shown in our GSEA analysis suggest that the effects of CHRDL2 in our system work directly through inhibition of BMP, it is not possible to rule out that some pathways are affected by BMP‐independent actions of CHRLD2. Indeed, Wang et al. suggest that CHRDL2 can directly alter phosphorylation and activity of YAP in gastric cancer cell lines, which merits further exploration (Wang et al., 2022). These data provide new avenues of research into the mechanism that CHRDL2 and potentially other BMP antagonists may exert their effects. Unravelling the pathways modulated by CHRDL2 and other BMP antagonists will undoubtedly drive future investigations in cancer research.

## 5 Conclusions

Our findings suggest that CHRDL2 should be further explored as a potential biomarker for increased chemotherapy resistance in CRC. This would necessitate a deeper understanding of the mechanism of resistance and also accurate determination of the pattern of overexpression of CHRDL2 during tumorigenesis and therapy. In summary, our data strongly suggest that CHRDL2, by inhibiting BMP signalling and augmenting WNT signalling, promotes stem‐cell properties in cancer cells, thus contributing to cancer progression and potentially therapeutic resistance.

## Conflict of interest

The authors declare no conflict of interest.

## Author contributions

AL and EC conceived the study. EC carried out cell line and organoid studies and carried out RNA‐seq analysis. AL provided resources and expertise. EC and AL wrote the manuscript.

## Peer review

The peer review history for this article is available at https://www.webofscience.com/api/gateway/wos/peer‐review/10.1002/1878‐0261.70064.

## Supporting information


**Fig. S1.** Quantification of CHRDL2, BMPs and P‐SMAD1/4 in parental and CHRDL2 overexpressing cell lines.
**Fig. S2.** Quantification of cell cycle analysis, cell proliferation by Ki67, clonogenic assays and IQGAP1 expression.
**Fig. S3.** Summary of IC50 values for CHRDL2 overexpressing cells treated with chemotherapy.
**Fig. S4.** Quantification of P‐SMAD1/5 protein, Ki67 immuno‐fluorescence and flow analysis of COLO320 cells, and Ku70 immunofluorescence analysis in CHRDL2 overexpressing cells treated with chemotherapy.
**Fig. S5.** Immunofluorescence based analysis of DNA damage repair pathway proteins in CHRDL2 overexpressing cells treated with chemotherapy.
**Fig. S6.** GSEA plots identifying disrupted pathways in CHRDL2 overexpressing cells as assessed by RNA sequencing.
**Fig. S7.** PANTHER Overrepresentation Test/GO Ontology analysis of RNA sequencing data from CHRDL2 overexpressing cells.

## Data Availability

The data are available within the article and/or the Supporting Information. The RNA sequencing raw data and associated files have been deposited on the Gene Expression Omnibus GSE253554.

## References

[1] Sung H , Ferlay J , Siegel RL , Laversanne M , Soerjomataram I , Jemal A , et al. Global cancer statistics 2020: GLOBOCAN estimates of incidence and mortality worldwide for 36 cancers in 185 countries. CA Cancer J Clin. 2021;71:209–249. 10.3322/caac.21660 33538338

[2] Jackstadt R , Hodder MC , Sansom OJ . WNT and β‐catenin in cancer: genes and therapy. Annu Rev Cancer Biol. 2020;4:177–196. 10.1146/annurev-cancerbio-030419-033628

[3] Gehart H , Clevers H . Tales from the crypt: new insights into intestinal stem cells. Nat Rev Gastroenterol Hepatol. 2019;16:19–34. 10.1038/s41575-018-0081-y 30429586

[4] Moore N , Lyle S . Quiescent, slow‐cycling stem cell populations in cancer: a review of the evidence and discussion of significance. J Oncol. 2011;2011:1–11. 10.1155/2011/396076 PMC2948913 20936110

[5] Sangiorgi E , Capecchi MR . Bmi1 is expressed in vivo in intestinal stem cells. Nat Genet. 2008;40:915–920. 10.1038/ng.165 18536716 PMC2906135

[6] Rees WD , Tandun R , Yau E , Zachos NC , Steiner TS . Regenerative intestinal stem cells induced by acute and chronic injury: the saving grace of the epithelium? Front Cell Dev Biol. 2020;8:583919. 10.3389/fcell.2020.583919 33282867 PMC7688923

[7] Nusse R , Clevers H . Wnt/β‐catenin signaling, disease, and emerging therapeutic modalities. Cell. 2017;169:985–999. 10.1016/j.cell.2017.05.016 28575679

[8] Batlle E , Henderson JT , Beghtel H , van den Born MMW , Sancho E , Huls G , et al. Β‐Catenin and TCF mediate cell positioning in the intestinal epithelium by controlling the expression of EphB/EphrinB. Cell. 2002;111:251–263. 10.1016/S0092-8674(02)01015-2 12408869

[9] Pinto D , Gregorieff A , Begthel H , Clevers H . Canonical Wnt signals are essential for homeostasis of the intestinal epithelium. Genes Dev. 2003;17:1709–1713. 10.1101/gad.267103 12865297 PMC196179

[10] Clevers H , Nusse R . Wnt/β‐catenin signaling and disease. Cell. 2012;149:1192–1205. 10.1016/j.cell.2012.05.012 22682243

[11] Kosinski C , Li VSW , Chan ASY , Zhang J , Ho C , Tsui WY , et al. Gene expression patterns of human colon tops and basal crypts and BMP antagonists as intestinal stem cell niche factors. Proc Natl Acad Sci USA. 2007;104:15418–15423. 10.1073/pnas.0707210104 17881565 PMC2000506

[12] Haramis A‐PG , Begthel H , van den Born M , van Es J , Jonkheer S , Offerhaus GJ , et al. De novo crypt formation and juvenile polyposis on BMP inhibition in mouse intestine. Science. 2004;303(5664):1684–1686. 10.1126/science.1093587 15017003

[13] Bertrand FE , Angus CW , Partis WJ , Sigounas G . Developmental pathways in colon cancer. Cell Cycle. 2012;11:4344–4351. 10.4161/cc.22134 23032367 PMC3552917

[14] Ye L , Kynaston H , Jiang WG . Bone morphogenetic Protein‐9 induces apoptosis in prostate cancer cells, the role of prostate apoptosis Response‐4. Mol Cancer Res. 2008;6:1594–1606. 10.1158/1541-7786.MCR-08-0171 18922975

[15] Wang L , Park P , Zhang H , la Marca F , Claeson A , Valdivia J , et al. BMP‐2 inhibits the tumorigenicity of cancer stem cells in human osteosarcoma OS99‐1 cell line. Cancer Biol Ther. 2011;11:457–463. 10.4161/cbt.11.5.14372 21178508 PMC3230314

[16] Fukuda T , Fukuda R , Tanabe R , Koinuma D , Koyama H , Hashizume Y , et al. BMP signaling is a therapeutic target in ovarian cancer. Cell Death Dis. 2020;6:139. 10.1038/s41420-020-00377-w PMC7719168 33298901

[17] Peng J , Yoshioka Y , Mandai M , Matsumura N , Baba T , Yamaguchi K , et al. The BMP signaling pathway leads to enhanced proliferation in serous ovarian cancer‐a potential therapeutic target. Mol Carcinog. 2016;55:335–345. 10.1002/mc.22283 25663289

[18] Hardwick JC , Kodach LL , Offerhaus GJ , Van den Brink GR . Bone morphogenetic protein signalling in colorectal cancer. Nat Rev Cancer. 2008;8:806–812. 10.1038/nrc2467 18756288

[19] Tzavlaki K , Moustakas A . TGF‐β signaling. Biomolecules. 2020;10(3):487. 10.3390/biom10030487 32210029 PMC7175140

[20] Walsh DW , Godson C , Brazil DP , Martin F . Extracellular BMP‐antagonist regulation in development and disease: tied up in knots. Trends Cell Biol. 2010;20:244–256. 10.1016/j.tcb.2010.01.008 20188563

[21] Sharov AA , Mardaryev AN , Sharova TY , Grachtchouk M , Atoyan R , Byers HR , et al. Bone morphogenetic protein antagonist noggin promotes skin tumorigenesis via stimulation of the Wnt and shh signaling pathways. Am J Pathol. 2009;175:1303–1314. 10.2353/ajpath.2009.090163 19700758 PMC2731148

[22] Ouahoud S , Hardwick JCH , Hawinkels LJAC . Extracellular BMP antagonists, multifaceted orchestrators in the tumor and its microenvironment. Int J Mol Sci. 2020;21:3888. 10.3390/ijms21113888 32486027 PMC7313454

[23] Kobayashi H , Gieniec KA , Wright JA , Wang T , Asai N , Mizutani Y , et al. The balance of stromal BMP signaling mediated by GREM1 and ISLR drives colorectal carcinogenesis. Gastroenterology. 2021;160:1224–1239. 10.1053/j.gastro.2020.11.011 33197448 PMC7617122

[24] Berglar I , Hehlgans S , Wehle A , Roth C , Herold‐Mende C , Rödel F , et al. CHRDL1 regulates stemness in glioma stem‐like cells. Cells. 2022;11:3917. 10.3390/cells11233917 36497175 PMC9741078

[25] Davis H , Irshad S , Bansal M , Rafferty H , Boitsova T , Bardella C , et al. Aberrant epithelial GREM1 expression initiates colonic tumorigenesis from cells outside the stem cell niche. Nat Med. 2015;21:62–70. 10.1038/nm.3750 25419707 PMC4594755

[26] Li D , Xie X‐Y , Shen H , Yuan ST , Liu QH , Yao Y . Chordin‐like 2 influences the differentiation fate of retinal pigment epithelium cells by dynamically regulating BMP pathway. Int J Ophthalmol. 2022;15(5):711–720. 10.18240/ijo.2022.05.04 35601169 PMC9091882

[27] Sun J , Liu X , Gao H , Zhang L , Ji Q , Wang Z , et al. Overexpression of colorectal cancer oncogene CHRDL2 predicts a poor prognosis. Oncotarget. 2017;8:11489–11506. 10.18632/oncotarget.14039 28009989 PMC5355280

[28] Wu I , Moses MA . BNF‐1, a novel gene encoding a putative extracellular matrix protein, is overexpressed in tumor tissues. Gene. 2003;311:105–110. 10.1016/S0378-1119(03)00563-8 12853144

[29] Sun J , Zhao J , Jiang F , Wang L , Xiao Q , Han F , et al. Identification of novel protein biomarkers and drug targets for colorectal cancer by integrating human plasma proteome with genome. Genome Med. 2023;15:75. 10.1186/s13073-023-01229-9 37726845 PMC10508028

[30] Chen H , Pan R , Li H , Zhang W , Ren C , Lu Q , et al. CHRDL2 promotes osteosarcoma cell proliferation and metastasis through the BMP‐9/PI3K/AKT pathway. Cell Biol Int. 2021;45:623–632. 10.1002/cbin.11507 33245175 PMC8049056

[31] Hebert JD , Tian C , Lamar JM , Rickelt S , Abbruzzese G , Liu X , et al. The scaffold protein IQGAP1 is crucial for extravasation and metastasis. Sci Rep. 2020;10:2439. 10.1038/s41598-020-59438-w 32051509 PMC7015931

[32] Van der Jeught K , Xu H‐C , Li Y‐J , Jeught K , Lu X‐B , Ji G . Drug resistance and new therapies in colorectal cancer. World J Gastroenterol. 2018;24(34):3834–3848. 10.3748/wjg.v24.i34.3834 30228778 PMC6141340

[33] Arango D , Wilson AJ , Shi Q , Corner GA , Arañes MJ , Nicholas C , et al. Molecular mechanisms of action and prediction of response to oxaliplatin in colorectal cancer cells. Br J Cancer. 2004;91:1931–1946. 10.1038/sj.bjc.6602215 15545975 PMC2409767

[34] Sato T , Vries RG , Snippert HJ , van de Wetering M , Barker N , Stange DE , et al. Single Lgr5 stem cells build crypt‐villus structures in vitro without a mesenchymal niche. Nature. 2009;459:262–265. 10.1038/nature07935 19329995

[35] van der Flier LG , Haegebarth A , Stange DE , van de Wetering M , Clevers H . OLFM4 is a robust marker for stem cells in human intestine and Marks a subset of colorectal cancer cells. Gastroenterology. 2009;137:15–17. 10.1053/j.gastro.2009.05.035 19450592

[36] Beumer J , Puschhof J , Yengej FY , Zhao L , Martinez‐Silgado A , Blotenburg M , et al. BMP gradient along the intestinal villus axis controls zonated enterocyte and goblet cell states. Cell Rep. 2022;38:110438. 10.1016/j.celrep.2022.110438 35235783

[37] Song Y , Bi Z , Liu Y , Qin F , Wei Y , Wei X . Targeting RAS–RAF–MEK–ERK signaling pathway in human cancer: current status in clinical trials. Genes Dis. 2023;10:76–88. 10.1016/j.gendis.2022.05.006 37013062 PMC10066287

[38] Cheng X , Xu X , Chen D , Zhao F , Wang W . Therapeutic potential of targeting the Wnt/β‐catenin signaling pathway in colorectal cancer. Biomed Pharmacother. 2019;110:473–481. 10.1016/j.biopha.2018.11.082 30530050

[39] Law PJ , Timofeeva M , Fernandez‐Rozadilla C , Broderick P , Studd J , Fernandez‐Tajes J , et al. Association analyses identify 31 new risk loci for colorectal cancer susceptibility. Nat Commun. 2019;10:2154. 10.1038/s41467-019-09775-w 31089142 PMC6517433

[40] Dariya B , Aliya S , Merchant N , Alam A , Nagaraju GP . Colorectal cancer biology, diagnosis, and therapeutic approaches. Crit Rev Oncog. 2020;25:71–94. 10.1615/CritRevOncog.2020035067 33389859

[41] Sobrero A , Guglielmi A , Grossi F , Puglisi F , Aschele C . Mechanism of action of fluoropyrimidines: relevance to the new developments in colorectal cancer chemotherapy. Semin Oncol. 2000;27:72–77. 11049035

[42] Srinivas US , Dyczkowski J , Beißbarth T , Gaedcke J , Mansour WY , Borgmann K , et al. 5‐fluorouracil sensitizes colorectal tumor cells towards double stranded DNA breaks by interfering with homologous recombination repair. Oncotarget. 2015;6:12574–12586. 10.18632/oncotarget.3728 25909291 PMC4494959

[43] Phi LTH , Sari IN , Yang Y‐G , Lee SH , Jun N , Kim KS , et al. Cancer stem cells (CSCs) in drug resistance and their therapeutic implications in cancer treatment. Stem Cells Int. 2018;2018:1–16. 10.1155/2018/5416923 PMC5850899 29681949

[44] Abdullah LN , Chow EK . Mechanisms of chemoresistance in cancer stem cells. Clin Transl Med. 2013;2(1):3. 10.1186/2001-1326-2-3 23369605 PMC3565873

[45] Zhou H‐M , Zhang J‐G , Zhang X , Li Q . Targeting cancer stem cells for reversing therapy resistance: mechanism, signaling, and prospective agents. Signal Transduct Target Ther. 2021;6:62. 10.1038/s41392-020-00430-1 33589595 PMC7884707

[46] Tao S , Tang D , Morita Y , Sperka T , Omrani O , Lechel A , et al. Wnt activity and basal niche position sensitize intestinal stem and progenitor cells to DNA damage. EMBO J. 2015;34:624–640. 10.15252/embj.201490700 25609789 PMC4365032

[47] Sheng X , Lin Z , Lv C , Shao C , Bi X , Deng M , et al. Cycling stem cells are radioresistant and regenerate the intestine. Cell Rep. 2020;32:107952. 10.1016/j.celrep.2020.107952 32726617 PMC7789978

[48] Montgomery RK , Carlone DL , Richmond CA , Farilla L , Kranendonk MEG , Henderson DE , et al. Mouse telomerase reverse transcriptase (mTert) expression marks slowly cycling intestinal stem cells. Proc Natl Acad Sci USA. 2011;108:179–184. 10.1073/pnas.1013004108 21173232 PMC3017192

[49] Koppens MAJ , Davis H , Valbuena GN , Mulholland EJ , Nasreddin N , Colombe M , et al. Bone morphogenetic protein pathway antagonism by Grem1 regulates epithelial cell fate in intestinal regeneration. Gastroenterology. 2021;161:239–254.e9. 10.1053/j.gastro.2021.03.052 33819486 PMC7613733

