# Supplementary material for: BMP antagonist CHRDL2 enhances the cancer stem‐cell phenotype and increases chemotherapy resistance in colorectal cancer
Source: Mol Oncol. 2025 May 28;19(11):3135–55. doi: 10.1002/1878-0261.70064 (PMC12591322; doi:10.1002/1878-0261.70064)
Supplement: Supplementary file 1 — Fig. S1. Quantification of CHRDL2, BMPs and P‐SMAD1/4 in parental and CHRDL2 overexpressing cell lines. Fig. S2. Quantification of cell cycle analysis, cell proliferation by Ki67, clonogenic assays and IQGAP1 expression. Fig. S3. Summary of IC50 values for CHRDL2 overexpressing cells treated with chemotherapy. Fig. S4. Quantification of P‐SMAD1/5 protein, Ki67 immuno‐fluorescence and flow analysis of COLO320 cells, and Ku70 immunofluorescence analysis in CHRDL2 overexpressing cells treated with chemotherapy. Fig. S5. Immunofluorescence based analysis of DNA damage repair pathway proteins in CHRDL2 overexpressing cells treated with chemotherapy. Fig. S6. GSEA plots identifying disrupted pathways in CHRDL2 overexpressing cells as assessed by RNA sequencing. Fig. S7. PANTHER Overrepresentation Test/GO Ontology analysis of RNA sequencing data from CHRDL2 overexpressing cells. [file MOL2-19-3135-s001.pdf]

Supplementary 1: Quantification of CHRDL2, BMPs and P-SMAD1/4 in parental and CHRDL2 overexpressing cell lines.

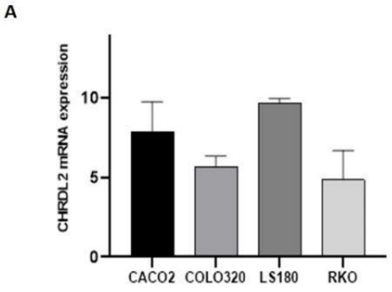

B

Cell Line	APC mutations	BMP receptor mutations	β-catenin mutations
CACO2	APC- Damaging		CTNNB1- Damaging
COLO320	APC- Damaging		
LS180	APC- Non-conserving	BMPR2- Damaging	CTNNB1- Damaging
RKO	APC2- Non-conserving	BMPR2- Damaging<br>BMPR1- Non-conserving	

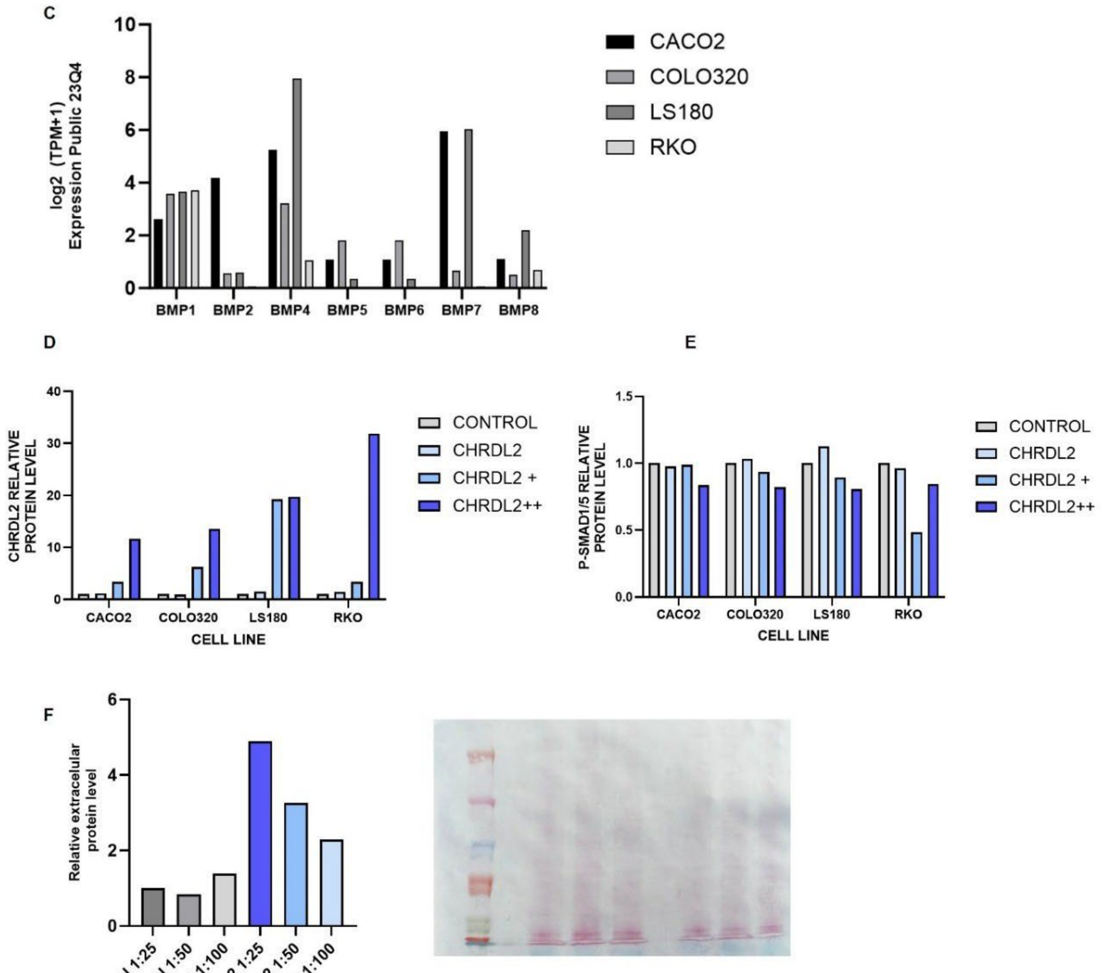

Supplementary figure 1: A) qPCR of endogenous CHRDL2 expression in experimental cell lines N=3. B) Table of known WNT pathway mutations in experimental cell lines Data obtained from the Cancer cell line encyclopaedia (CCLE). C) Levels of endogenous BMP proteins in experimental cell lines given as log2 (TPM + 1). D) Quantification of CHRDL2 protein in experimental cell lines N=2. E) Quantification of secreted CHRDL2 by RKO CHRDL2 overexpressing cells Ponceau stain was used as a loading control N=2. F) Quantification of P-SMAD1/5 protein levels in CRC cells with CHRDL2 overexpression N=3. Error bars given as ± SEM.

Supplementary 2:

Quantification of cell cycle analysis, cell proliferation by Ki67, clonogenic assays and IQGAP1 expression

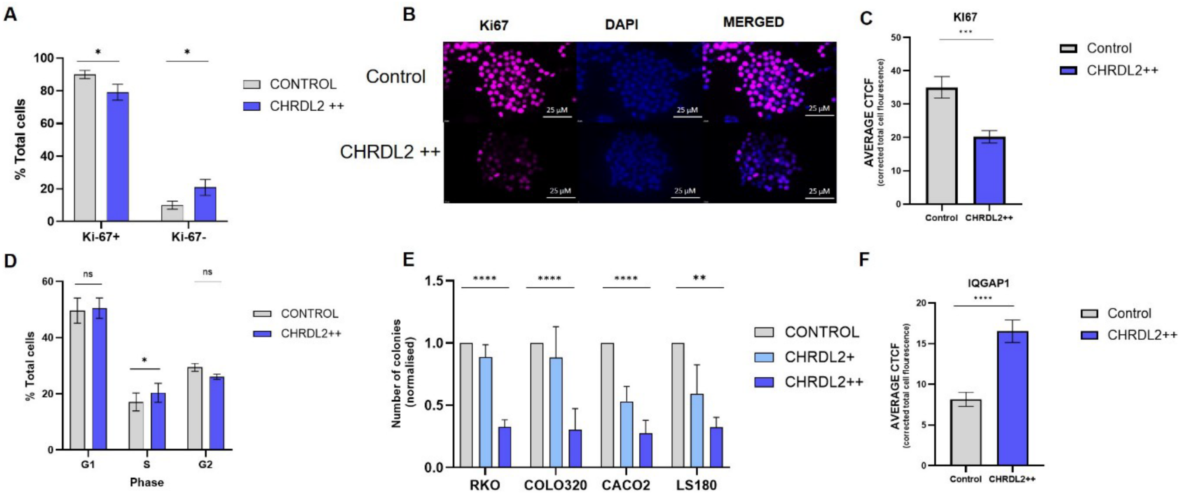

Supplementary figure 2: A) Quantification of Ki67+/- cells by flow cytometry in COLO320 control and CHRDL2++ cells, T-test P<0.0485. N=3 B) Immunofluorescence of Ki67 expression in COLO320 CHRDL2+ cells compared to a control. Representative images N=3 C) Quantification of Ki67 immunofluorescence staining on COLO320 CHRDL2+ cells compared to a control, T-test P<0.005. N=3. D) Quantification of cell cycle status by flow cytometry in COLO320 control and CHRDL2++ cells, T-test p=0.0076 N=3. E) Quantification of clonogenic colonies established in our 4 experimental cell lines with CHRDL2 overexpression. CACO2 and RKO cell lines both showed reduced colony formation in the low and high CHRDL2 treated groups, T-test: RKO p<0.01, COLO320 p<0.001, CACO2 p<0.05, LS180 P<0.001, T-test N=3. COLO320 and LS180 both showed a reduction in colony formation in the high CHRDL2 group only, p<0.01. F) Quantification of IQGAP1 immunofluorescence staining on COLO320 CHRDL2+ cells compared to a control. T-test P<0.0005. N=3. In all panels * = p<0.05, ** = p<0.01, *** = p<0.001, **** = p<0.0001, ns = p>0.05. Error bars are given as ± SEM. Scale bar indicates 25 μM.

Supplementary 3: Summary of IC50 values for CHRDL2 overexpressing cells treated with chemotherapy.

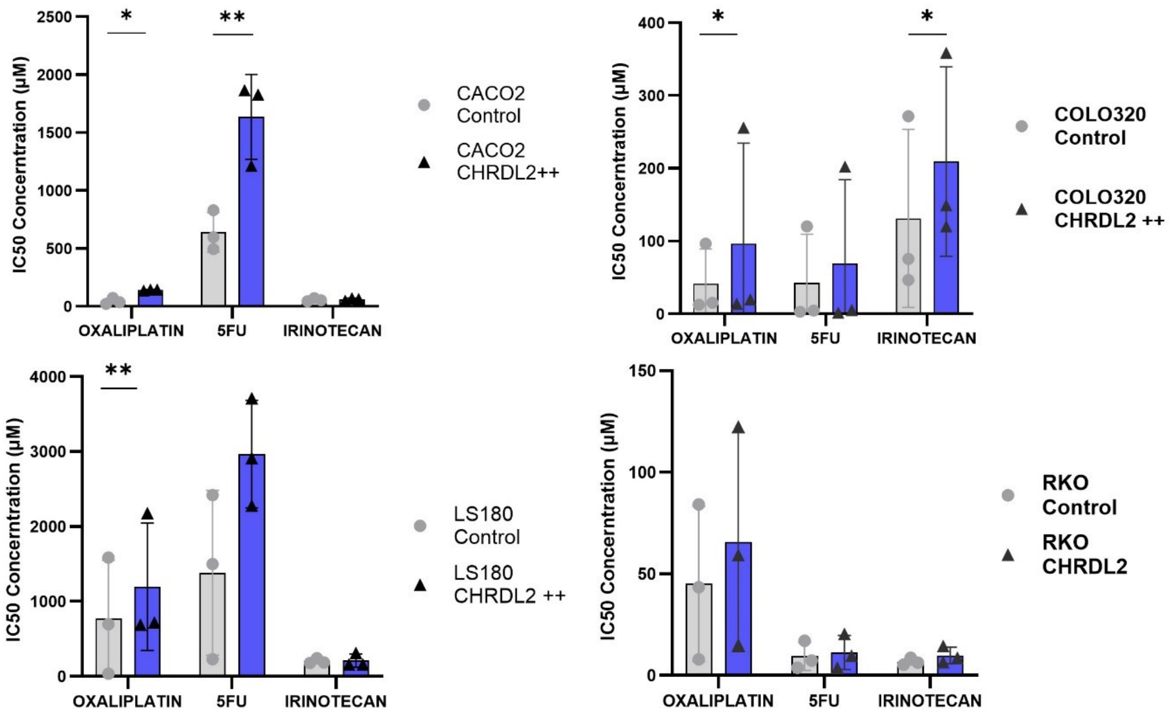

Supplementary figure 3: Average IC50 values of CACO2, COLO320, LS180 and RKO cell lines using chemotherapy drugs Oxaliplatin, 5FU, and Irinotecan N=3. T-test: CACO2 Oxaliplatin $P<0.05$ 5FU $P<0.01$. COLO320 Oxaliplatin $P<0.05$, Irinotecan $P<0.05$. LS180 Oxaliplatin $P<0.01$. In all panels * = $p<0.05$, ** = $p<0.01$, ns = $p>0.05$. Error bars given as ± SEM.

Supplementary 4: Quantification of P-SMAD1/5 protein, Ki67 immuno-fluorescence and flow analysis of COLO320 cells, and Ku70 immunofluorescence analysis in CHRDL2 overexpressing cells treated with chemotherapy.

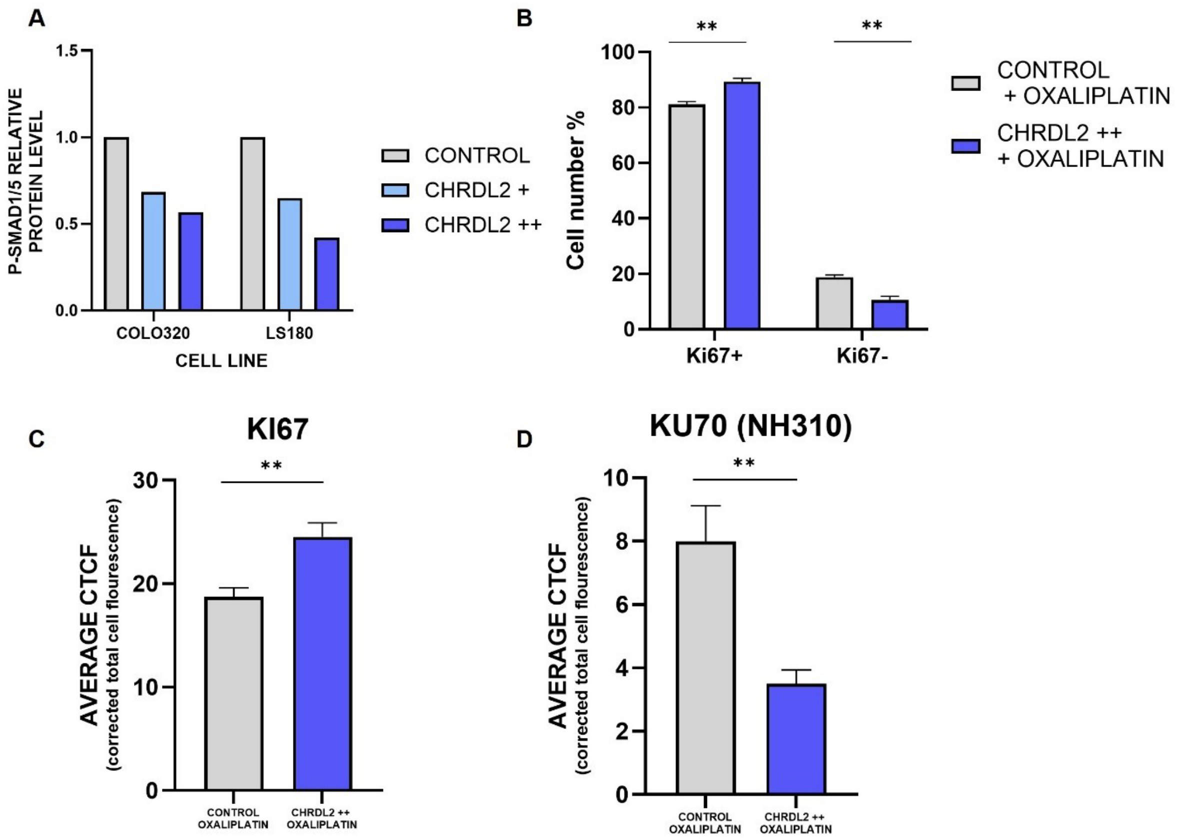

Supplementary figure 4: A) Quantification of P-SMAD1/5 protein level analysed by western blot in COLO320 and Ls180 cells treated with IC50 5FU chemotherapy. B) Flow cytometry analysis of Ki67 expression by COLO320 cells treated with 5 μM oxaliplatin (IC25). T-test. P<0.0056, p<0.0055. N=3. C) Quantification of Ki67 in COLO320 cells overexpressing CHRDL2 treated with 5 μM oxaliplatin 72 hrs. Cells were treated with DMSO control reagent, or Doxycycline to induce CHRDL2 overexpression. T-test P<0.0064. N=3 D) Quantification of Ku70 in COLO320 cells overexpressing CHRDL2 treated with 5 μM oxaliplatin 72 hrs. Cells were treated with DMSO control reagent, or Doxycycline to induce CHRDL2 overexpression. T-test P<0.0057 N=3. In all panels * = p<0.05, ** = p<0.01, Error bars given as ± SEM.

Supplementary 5: Immunofluorescence based analysis of DNA damage repair pathway proteins in CHRDL2 overexpressing cells treated with chemotherapy.

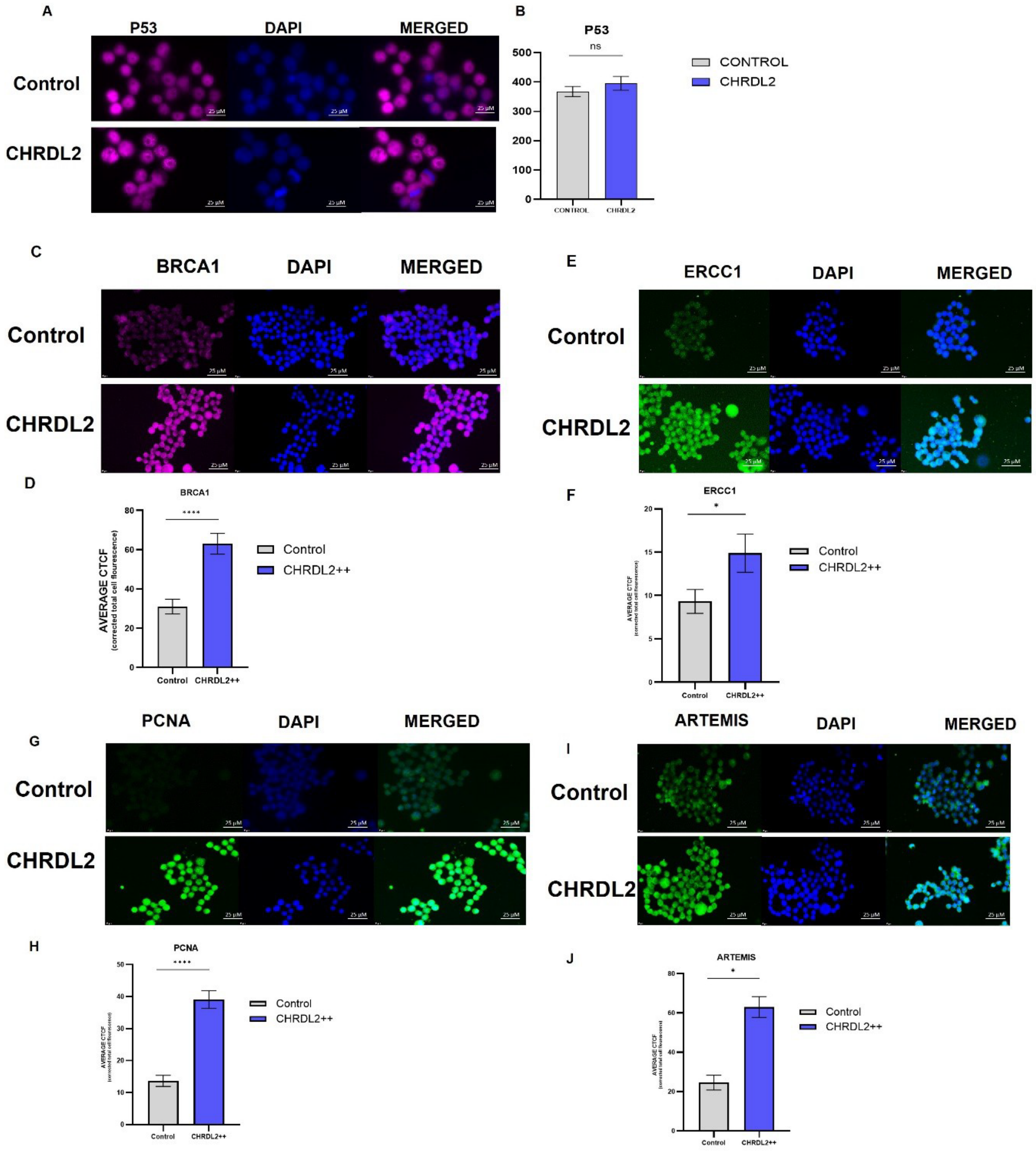

Supplementary figure 5: A) Immunofluorescence staining of P53 on COLO320 cells treated with 5 μM oxaliplatin at 72 hrs Representative images N=3. B) Quantification of P53 in COLO320 cells overexpressing CHRDL2 treated with 5 μM oxaliplatin 72 hrs. Cells were treated with DMSO control reagent, or Doxycycline to induce CHRDL2 overexpression N=3. T-test C) Immunofluorescence staining of BRCA1 on COLO320 cells treated with 5 μM oxaliplatin. Representative images N=3 D) Quantification of BRCA1 in COLO320 cells. Immunofluorescence given as Corrected Total Cell Fluorescence (CTCF). Cells were treated with DMSO control reagent, or Doxycycline to induce CHRDL2++ overexpression. P<0.0001 N=3 T-test. E) Immunofluorescence staining of ERCC1 on COLO320 cells treated with 5 μM oxaliplatin. Representative images N=3 F) Quantification of ERCC1 in COLO320 cells. Immunofluorescence given as Corrected Total Cell Fluorescence (CTCF). Cells were treated with DMSO control reagent, or Doxycycline to induce CHRDL2++ overexpression. P<0.05 N=3 T-test. G) Immunofluorescence staining of PCNA on COLO320 cells treated with 5 μM oxaliplatin. Representative images N=3 H) Quantification of PCNA in COLO320 cells. Immunofluorescence given as Corrected Total Cell Fluorescence (CTCF). Cells were treated with DMSO control reagent, or Doxycycline to induce CHRDL2++ overexpression. P<0.0001 N=3 T-test. I) Immunofluorescence staining of ARTEMIS on COLO320 cells treated with 5 μM oxaliplatin. Representative images N=3 J) Quantification of ARTEMIS in COLO320 cells. Immunofluorescence given as Corrected Total Cell Fluorescence (CTCF). Cells were treated with DMSO control reagent, or Doxycycline to induce CHRDL2++ overexpression. P<0.05 N=3 T-test. In all panels ns = non-significant * = p<0.05, **** = p<0.0001, ns = p>0.05. Error bars given as ± SEM. Scale bar indicates 25 μM.

Supplementary 6: GSEA plots identifying disrupted pathways in CHRDL2 overexpressing cells as assessed by RNA sequencing.

WNT Signalling

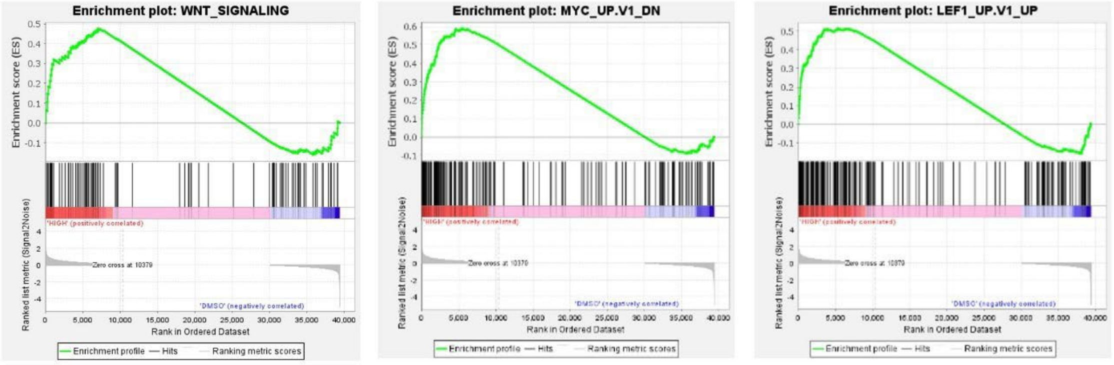

YAP Signalling

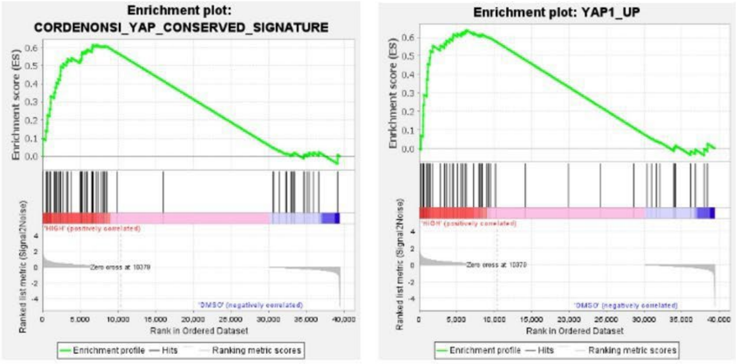

BMI1 Signalling

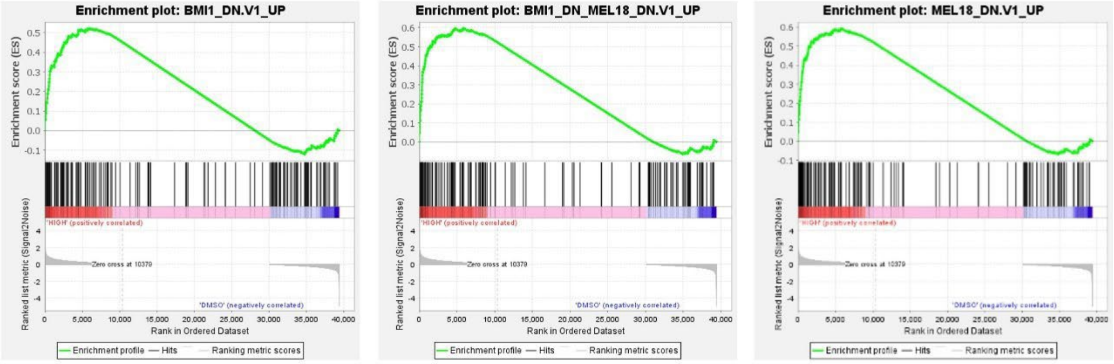

RAF Signalling

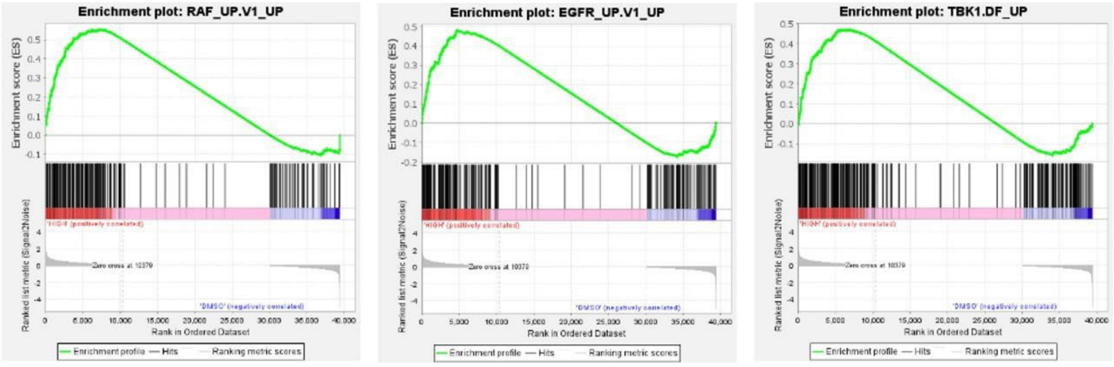

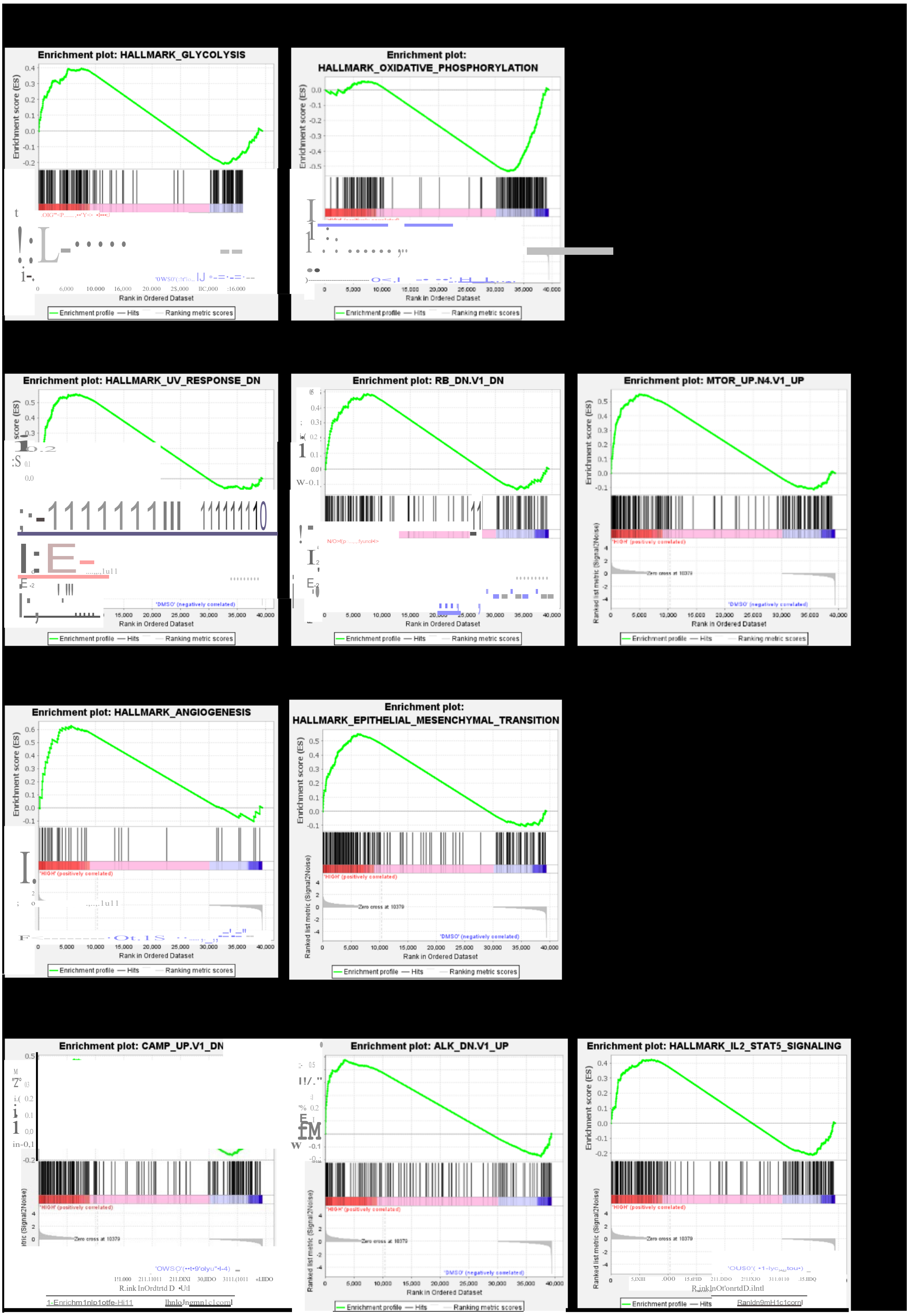
Enrichment plot: HALLMARK_GLYCOLYSIS
Enrichment plot: HALLMARK_OXIDATIVE_PHOSPHORYLATION
Enrichment plot: HALLMARK_UV_RESPONSE_DN
Enrichment plot: RB_DN.V1_DN
Enrichment plot: MTOR_UP.N4.V1_UP
Enrichment plot: HALLMARK_ANGIOGENESIS
Enrichment plot: HALLMARK_EPITHELIAL_MESENCHYMAL_TRANSITION
Enrichment plot: CAMP_UP.V1_DN
Enrichment plot: ALK_DN.V1_UP
Enrichment plot: HALLMARK_IL2_STAT5_SIGNALING
Enrichment score (ES)
Ranked list metric (Signal2Noise)
Rank in Ordered Dataset
'HIGH' (positively correlated)
'DMSO' (negatively correlated)
Zero cross at 10379
Enrichment profile — Hits
Ranking metric scores

Supplementary figure 6: GSEA plots of RNAseq analysis from CHRDL2++ cells (HIGH) compared to DMSO control. P values given as FWER. WNT_SIGNALLING NES= 1.15 P<0.0. MYC_UP.V1_DN NES=1.25 P<0.01. LEF1_UP_V1_UP NES=1.26 P<0.01. CORDENOSI_YAP_CONSERVED_SIGNATURE NES=1.36 P<0.01. YAP1_UP NES=1.32 P<0.01. BMI1_DN.V1_UP NES=1.33 P<0.01. BMI1_DN_MEL18_DN.V1_UP NES=1.38 P<0.01. MEL18_DN.V1_UP NES=1.41 P<0.0. RAF_UP.V1_UP NES=1.2 P<0.01. EGFR_UP.V1_UP NES= 1.25 P<0.0. TBK1.DF_UP NES=1.2 P<0.01. HALLMARK_GLYCOLYSIS NES=1,13 P<0.01. HALLMARK_OXIDATIVE_PHOSPHORYLATION NES=-1.1 P<0.01. HALLMARK_UV_RESPONSE_DN NES=1.199 P<0.01. RB_DN.V1_DN NES=1.2 P<0.01. MTOR_UP.N4.V1_UP NES=1.31 P<0.01. HALLMARK_ANGIOGENESIS NES=1.21 P<0.01. HALLMARK_MESENCHYMAL_TRANSITION NES=1.29 P<0.01. CAMP_UP.V1_DN NES=1.18 P<0.01. ALK_DN.V1_UP NES=1.36 P<0.01. HALLMARK_IL2_STAT5_SIGNALLING NES=1.15 P<0.01. N=3.

Supplementary 7: PANTHER Overrepresentation Test/ GO Ontology analysis of RNA sequencing data from CHRDL2 overexpressing cells

GO biological process complete	Homo sapiens – REFLIST (20592)	DIFFENTIALLY EXPRESSED BY CHRDL2 (130)	over/under	fold Enrichment	raw P-value	FDR
triglyceride mobilization (GO:0006642)	3	2	+	> 100	1.18E-04	4.29E-02
neutrophil homeostasis (GO:0001780)	17	3	+	27.95	1.57E-04	4.87E-02
positive regulation of substrate adhesion-dependent cell spreading (GO:1900026)	43	4	+	14.73	1.55E-04	5.02E-02
proteinogenic amino acid biosynthetic process (GO:0170038)	54	5	+	14.67	2.29E-05	2.05E-02
L-amino acid biosynthetic process (GO:0170034)	54	5	+	14.67	2.29E-05	1.94E-02
cellular response to glucocorticoid stimulus (GO:0071385)	55	5	+	14.4	2.51E-05	2.01E-02
cellular response to corticosteroid stimulus (GO:0071384)	65	5	+	12.18	5.66E-05	3.32E-02
alpha-amino acid biosynthetic process (GO:1901607)	65	5	+	12.18	5.66E-05	3.20E-02
amino acid biosynthetic process (GO:0008652)	67	5	+	11.82	6.55E-05	3.12E-02
regulation of lipid biosynthetic process (GO:0046890)	175	7	+	6.34	1.25E-04	4.32E-02
regulation of small molecule metabolic process (GO:0062012)	324	11	+	5.38	6.80E-06	1.48E-02
carboxylic acid biosynthetic process (GO:0046394)	278	9	+	5.13	6.94E-05	3.21E-02
organic acid biosynthetic process (GO:0016053)	281	9	+	5.07	7.53E-05	3.19E-02
regulation of hormone levels (GO:0010817)	550	14	+	4.03	1.02E-05	1.29E-02
positive regulation of transport (GO:0051050)	855	18	+	3.33	7.24E-06	1.38E-02
carboxylic acid metabolic process (GO:0019752)	801	16	+	3.16	4.58E-05	3.04E-02
oxoacid metabolic process (GO:0043436)	823	16	+	3.08	6.31E-05	3.10E-02
organic acid metabolic process (GO:0006082)	830	16	+	3.05	6.98E-05	3.13E-02
response to hormone (GO:0009725)	785	15	+	3.03	1.31E-04	4.45E-02
positive regulation of developmental process (GO:0051094)	1337	25	+	2.96	8.50E-07	4.32E-03
positive regulation of cell differentiation (GO:0045597)	871	16	+	2.91	1.22E-04	4.33E-02
regulation of apoptotic process (GO:0042981)	1476	24	+	2.58	1.58E-05	1.72E-02
lipid metabolic process (GO:0006629)	1238	20	+	2.56	9.71E-05	3.79E-02
regulation of programmed cell death (GO:0043067)	1521	24	+	2.5	2.58E-05	1.97E-02
response to endogenous stimulus (GO:0009719)	1411	22	+	2.47	7.04E-05	3.07E-02
response to biotic stimulus (GO:0009607)	1429	22	+	2.44	8.48E-05	3.40E-02
response to other organism (GO:0051707)	1382	21	+	2.41	1.51E-04	4.99E-02
response to external biotic stimulus (GO:0043207)	1385	21	+	2.4	1.55E-04	4.93E-02
regulation of developmental process (GO:0050793)	2448	35	+	2.26	3.44E-06	1.05E-02
regulation of localization (GO:0032879)	2028	28	+	2.19	7.58E-05	3.12E-02
anatomical structure morphogenesis (GO:0009653)	2239	30	+	2.12	5.43E-05	3.45E-02
regulation of multicellular organismal process (GO:0051239)	2966	39	+	2.08	4.15E-06	1.05E-02
response to external stimulus (GO:0009605)	2290	30	+	2.08	1.06E-04	3.95E-02
regulation of biological quality (GO:0065008)	2849	37	+	2.06	1.62E-05	1.65E-02
response to organic substance (GO:0010033)	2467	32	+	2.05	5.91E-05	3.10E-02
cell differentiation (GO:0030154)	3654	44	+	1.91	8.65E-06	1.32E-02
cellular developmental process (GO:0048869)	3657	44	+	1.91	8.78E-06	1.22E-02
system development (GO:0048731)	3546	41	+	1.83	5.84E-05	3.18E-02
multicellular organism development (GO:0007275)	3964	45	+	1.8	4.41E-05	3.05E-02
anatomical structure development (GO:0048856)	5231	59	+	1.79	7.85E-07	5.98E-03
developmental process (GO:0032502)	5739	63	+	1.74	6.11E-07	9.32E-03
cell communication (GO:0007154)	5262	55	+	1.66	2.92E-05	2.12E-02
signal transduction (GO:0007165)	4789	50	+	1.65	9.84E-05	3.75E-02
signaling (GO:0023052)	5109	53	+	1.64	5.97E-05	3.03E-02
positive regulation of cellular process (GO:0048522)	5707	59	+	1.64	1.84E-05	1.75E-02
multicellular organismal process (GO:0032501)	6745	69	+	1.62	1.96E-06	7.46E-03
positive regulation of biological process (GO:0048518)	6210	63	+	1.61	1.26E-05	1.47E-02
cellular response to stimulus (GO:0051716)	6448	63	+	1.55	5.57E-05	3.40E-02
response to stimulus (GO:0050896)	8182	77	+	1.49	8.29E-06	1.40E-02

Supplementary figure 7: PANTHER Overrepresentation Test on CHRDL2++ differentially expressed genes. Provided by GO ontology database.
